# Standardized next-generation sequencing of immunoglobulin and T-cell receptor gene recombinations for MRD marker identification in acute lymphoblastic leukaemia; a EuroClonality-NGS validation study

**DOI:** 10.1038/s41375-019-0496-7

**Published:** 2019-06-26

**Authors:** Monika Brüggemann, Michaela Kotrová, Henrik Knecht, Jack Bartram, Myriam Boudjogrha, Vojtech Bystry, Grazia Fazio, Eva Froňková, Mathieu Giraud, Andrea Grioni, Jeremy Hancock, Dietrich Herrmann, Cristina Jiménez, Adam Krejci, John Moppett, Tomas Reigl, Mikael Salson, Blanca Scheijen, Martin Schwarz, Simona Songia, Michael Svaton, Jacques J. M. van Dongen, Patrick Villarese, Stephanie Wakeman, Gary Wright, Giovanni Cazzaniga, Frédéric Davi, Ramón García-Sanz, David Gonzalez, Patricia J. T. A. Groenen, Michael Hummel, Elizabeth A. Macintyre, Kostas Stamatopoulos, Christiane Pott, Jan Trka, Nikos Darzentas, Anton W. Langerak, on behalf of the EuroClonality-NGS working group

**Affiliations:** 1 Department of Hematology, University Hospital Schleswig-Holstein, Kiel, Germany; 2 CLIP - Childhood Leukaemia Investigation Prague, Department of Paediatric Haematology and Oncology, Second Faculty of Medicine, Charles University, University Hospital Motol, Prague, Czech Republic; 3 Department of Paediatric Haematology, Great Ormond Street Hospital, London, UK; 4 Department of Hematology, Hopital Pitié-Salpêtrière, Paris, France; 5 Central European Institute of Technology, Masaryk University, Brno, Czech Republic; 6 Centro Ricerca Tettamanti, University of Milano Bicocca, Monza, Italy; 7 CNRS, CRIStAL, Université Lille, Inria Lille, France; 8 Bristol Genetics Laboratory, Southmead Hospital, Bristol, UK; 9 Hospital Universitario de Salamanca-IBSAL, Salamanca, Spain; 10 Department of Pediatric Haematology, Bristol Royal Hospital for Children, Bristol, UK; 11 Department of Pathology, Radboud University Medical Center, Nijmegen, The Netherlands; 12 Department of Immunohematology and Blood Transfusion (IHB), Leiden University Medical Center, Leiden, The Netherlands; 13 Department of Hematology, APHP Necker-Enfants Malades and Paris Descartes University, Paris, France; 14 Centre for Cancer Research and Cell Biology, Queen’s University Belfast, Belfast, UK; 15 Insititute of Pathology, Charité – Universitätsmedizin Berlin, Berlin, Germany; 16 Institute of Applied Biosciences, Thessaloniki, Greece; 17 Department of Immunology, Laboratory Medical Immunology, Erasmus MC, University Medical Center, Rotterdam, The Netherlands

**Keywords:** Cancer genetics, Genetics research

## Abstract

Amplicon-based next-generation sequencing (NGS) of immunoglobulin (IG) and T-cell receptor (TR) gene rearrangements for clonality assessment, marker identification and quantification of minimal residual disease (MRD) in lymphoid neoplasms has been the focus of intense research, development and application. However, standardization and validation in a scientifically controlled multicentre setting is still lacking. Therefore, IG/TR assay development and design, including bioinformatics, was performed within the EuroClonality-NGS working group and validated for MRD marker identification in acute lymphoblastic leukaemia (ALL). Five EuroMRD ALL reference laboratories performed IG/TR NGS in 50 diagnostic ALL samples, and compared results with those generated through routine IG/TR Sanger sequencing. A central polytarget quality control (cPT-QC) was used to monitor primer performance, and a central in-tube quality control (cIT-QC) was spiked into each sample as a library-specific quality control and calibrator. NGS identified 259 (average 5.2/sample, range 0–14) clonal sequences vs. Sanger-sequencing 248 (average 5.0/sample, range 0–14). NGS primers covered possible IG/TR rearrangement types more completely compared with local multiplex PCR sets and enabled sequencing of bi-allelic rearrangements and weak PCR products. The cPT-QC showed high reproducibility across all laboratories. These validated and reproducible quality-controlled EuroClonality-NGS assays can be used for standardized NGS-based identification of IG/TR markers in lymphoid malignancies.

## Introduction

Specific antigen recognition by cells of the adaptive immune system (B cells, T cells) is mediated through receptors (immunoglobulin, IG, and T-cell receptor, TR) that are uniquely formed during immune development in bone marrow and thymus, respectively. Through recombination of IG/TR loci a diverse (polyclonal) repertoire of unique IG/TR receptors is created. In certain autoimmune diseases this repertoire is skewed (oligoclonal), whereas in lymphoid malignancies receptors are largely identical (monoclonal) [1–7]. IG/TR rearrangements thus form unique genetic biomarkers (molecular signatures) for studying immune cells for clinical, diagnostic and research applications [8–11]. Classically, methods for immunogenetic analysis mostly concern fragment analysis and Sanger-based sequencing. The introduction of NGS makes deeper analysis of IG/TR rearrangements possible, with impact on the main immunogenetic applications: clonality assessment, MRD detection, repertoire analysis [12–29].

The EuroClonality-NGS working group (euroclonalityngs.org; Supplementary Figure 1) has ample expertise in development, standardization and validation of IG/TR assays, to address the challenges in the translational research towards clinical application.

Here we report on the development and standardization (see also accompanying manuscript by Knecht et al. [30]) of novel amplicon-based IG/TR NGS assays between September 2012 and October 2017, via a total of 14 international coordination and evaluation meetings (Supplementary Table 1). This study focuses on IG/TR marker identification in lymphoid malignancies for subsequent MRD analysis, and their multicentre validation in acute lymphoblastic leukaemia (ALL). Assay optimizations and modifications for other applications of IG/TR NGS are partly still ongoing and will be reported in separate publications.

## Materials and methods

### General concept of assay design

With the objective of developing a universal amplicon-based NGS approach for IG/TR sequence analysis at the DNA level, applicable in all lymphoid malignancies, assays for multiple IG/TR loci were designed: IG heavy (IGH), IG kappa (IGK), TR beta (TRB), TR gamma (TRG) and TR delta (TRD), including complete and incomplete rearrangements whenever applicable. IG lambda (IGL) was excluded due to its limited complementarity to other IG loci and its reduced diversity. TR alpha (TRA) was excluded due to its high complexity, severely hampering a reasonable multiplex PCR approach at the DNA level.

The IGH locus is rearranged in two steps. After initial coupling of a single IGHD gene to an IGHJ gene, an IGHV gene is joined to the incomplete IGHD–IGHJ rearrangement, resulting in a complete IGHV–IGHJ rearrangement. For amplification of complete IGH rearrangements, primers located in the FR1, FR2 and FR3 regions were designed, but here we only discuss the FR1 assay for marker identification in ALL (for application of IGH-VJ-FR3 assay in clonality testing see accompanying manuscript by Scheijen et al. [31]). IGHD–IGHJ rearrangements were amplified in a separate multiplex PCR reaction. The IGK light chain locus is composed of functional IGKV and IGKJ genes, as well as the so-called kappa deleting element (Kde) that can rearrange to IGKV genes, or to a recombination signal sequence (RSS) in the IGKJ–IGKC intron, leading to functional inactivation of the IGK allele. The IGKV forward primers were designed to be used in combination with IGKJ and Kde reverse primers in one multiplex reaction, whereas a second PCR was developed for the forward intron RSS and reverse Kde primers.

The TRB locus also features a two-step process with initial formation of incomplete TRBD–TRBJ rearrangements followed by complete TRBV–TRBJ rearrangements. Incomplete and complete TRB rearrangements are detected in two separate multiplex PCR reactions. As TRG locus rearrangements are one-step VJ recombinations involving a limited number of TRGV and TRGJ genes, a single multiplex assay could be developed. Finally, in the TRD locus, complete VJ rearrangements are preceded by DD, VD and DJ rearrangements. In addition, certain TRAV genes can rearrange to both TRDJ and TRAJ, whereas TRDV–TRAJ rearrangements, usually involving TRAJ29, can also occur. All of these rearrangements were designed to be amplified in one multiplex PCR assay.

Both the design and further testing were coordinated by the respective ‘Target’ network leaders: IGH-VJ by C. Pott, Kiel and R. Garcia Sanz, Salamanca; IGH-DJ by F. Davi, Paris and K. Stamatopoulos, Thessaloniki; IGK-V/intron-IGKJ/Kde by P.J.T.A. Groenen, Nijmegen and A.W. Langerak, Rotterdam; TRB by M. Brüggemann, Kiel and M. Hummel, Berlin; TRG by G. Cazzaniga, Monza and J.J.M. van Dongen, Leiden; and TRD by E. Macintyre, Paris. Initial testing of each assay was performed by 2–3 experienced laboratories per target and final assays were validated for IG/TR marker identification in ALL in a multicentre setting. In addition, central quality control procedures were developed to monitor assay performance.

The bioinformatic platform ARResT/Interrogate [32], developed from the ground-up within the EuroClonality-NGS to assist with its multi-faceted activities, was further adapted for this study as described below.

### Primer design and technical validation of primer performance

Primers were designed to be gene-specific, but in case of allelic variants, degenerate primers were designed to avoid differential annealing in individuals with different allelic variants. For the same reason, single mismatches in the middle or at the 5′-end of the primer were accepted.

Primer3 [33], Primer Digital (PrimerDigital Ltd, Helsinki, Finland) MFEprimer-2.0 [34] and Oligo (Molecular Biology Insights, Inc., Colorado, USA) were used for checking primer specificity and multiplexing. Common primer design criteria were followed for all loci: primer melting temperature 57–63 °C; comparable size of final amplicon; primer length 20–24 nt; avoidance of primer dimers; minimal distance of 3′ primer end to the junctional region of, preferably, >10–15 bp to avoid false-negativity for rearrangements with larger nucleotide deletions from the germline sequence; avoidance of regions with known single nucleotide polymorphisms to allow identical primer annealing for all alleles of the respective V, D or J genes; targeting of, preferably, all V, D and J genes known to be rearranged plus the intronRSS and Kde regions for IGK.

Following in silico design, primers were first tested in monoplex and multiplex reactions using primary patient samples or cell lines with defined rearrangements. In occasional cases where no such samples were available, healthy tonsil or mononuclear DNA samples were employed. Oligoclonal template pools were then created from mixtures of rearranged cell lines and diagnostic samples with defined rearrangements covering many different V, D and/or J genes. Alternatively, for some loci, plasmid pools were produced, covering as many different rearrangements as possible. These multi-target pools allowed fine-tuning of reaction conditions and/or primer concentrations to assess comparable amplification efficiencies. This iterative process of testing also led to a reduction of primers if these appeared redundant. Further multicentre testing was performed with a limited number of monoclonal and poly/oligoclonal samples on different sequencing platforms, which allowed assessment of robustness of the primer mixes and protocols.

As assays were designed with the aim to be platform-independent, a two-step PCR was employed, that enabled switching of sequencing adaptors and to reduce the total number of primers even if many barcodes are necessary. Also, maximal amplicon lengths were defined with respect to the possible maximal sequencing read lengths of current sequencers. PCR conditions were optimized with the aim to find optimal conditions common for all reactions, thus allowing for parallel library preparation. Various numbers of PCR cycles in 1st and 2nd PCR, different polymerases and several library purification methods were tested and compared.

### Multicentre validation of assays for MRD marker identification in ALL

Five experienced laboratories tested the robustness and applicability of the optimized assays for NGS-based IG/TR marker identification in ALL in comparison to standard techniques. All laboratories (Bristol/London, Paris, Monza, Prague and Kiel) are members of the EuroMRD consortium and reference laboratories for ALL MRD analysis. Each of them performed NGS-based IG/TR MRD marker identification in 10 patients with B- or T-lineage ALL. A central standard operating procedure was strictly followed. The study was executed using the Illumina MiSeq (2 × 250 bp v2 kit). NGS analyses were performed fully in parallel to conventional PCR plus Sanger sequencing of clonal products following standard guidelines [11]. For a part of the cases with unexplained discrepant results between the two methods, allele-specific PCR assays (either for digital droplet PCR or real-time quantitative PCR) were designed to clarify if the respective clonal rearrangement represented the leukaemic bulk. EuroMRD guidelines were used to design and interpret allele-specific PCR assays [35, 36].

## Results

### Primer design and technical validation of primer performance

**Fig. 1:**
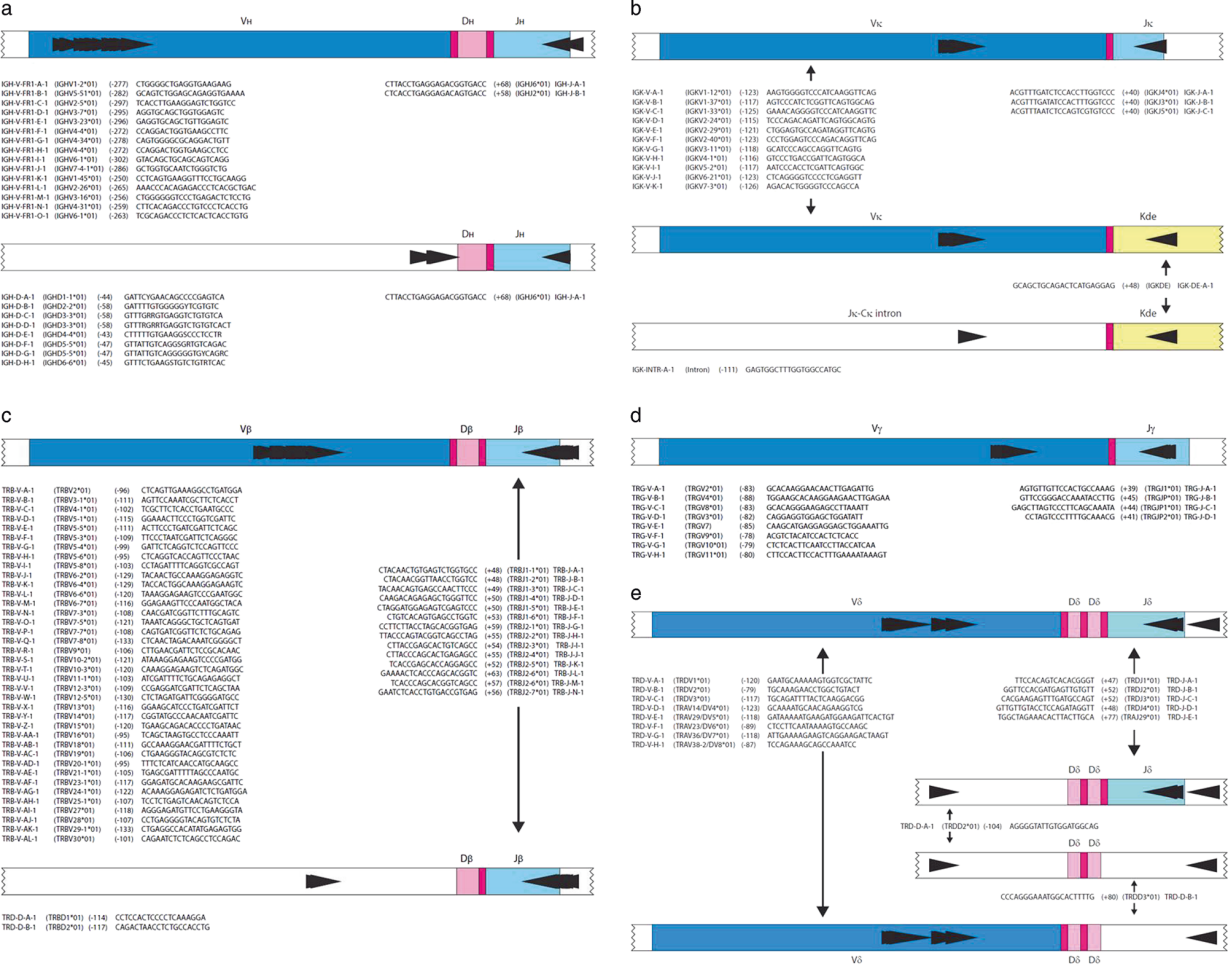
Schematic diagrams of rearrangements and primer sets. **a** Schematic diagrams of IGHV-IGHJ and IGHD-IGHJ rearrangements. The relative position of the VH family primers, DH family primers and consensus JH primers is given according to their most 5′ nucleotide upstream (−) or downstream (+) of the involved RSS. **b** Schematic diagrams of IGKV-IGKJ rearrangement and the two types of Kde rearrangements (V-Kde and intronRSS–Kde). The relative position of the IGKV, IGKJ, Kde, and intronRSS (INTR) primers is given according to their most 5′ nucleotide upstream (−) or downstream (+) of the involved RSS. **c** Schematic diagrams of TRBV-TRBJ rearrangement and TRBD-TRBJ rearrangement. The relative position of the TRBV family primers, TRBD primers and the TRBJ primers is given according to their most 5′nucleotide upstream (−) or downstream (+) of the involved RSS. **d** Schematic diagrams of TRGV–TRGJ rearrangement and the relative position of the TRGV and TRGJ primers. The relative position of the TRGV primers and the TRGJ primers is given according to their most 5′ nucleotide upstream (−) or downstream (+) of the involved RSS. **e** Schematic diagram of TRDV–TRDJ,TRDD–TRDJ, TRDD–TRDD, and TRDV–TRDD, TRDV-TRAJ29 rearrangements, showing the positioning of TRDV, TRDJ, TRDD, and TRAJ29 primers, all combined in a single tube. The relative position of the TRDV, TRDD, and TRDJ primers is indicated according to their most 50 nucleotides upstream (−) or downstream (+) of the involved RSS

Based on the results of the testing and validation phases (Supplementary Table 2), the final IG/TR primer mixes consisted of eight tubes with 92 forward and 30 reverse primers, 15 of the latter being used in pairs of different tubes (Supplementary Table 3). Primer positions and sequences are presented in Fig. 1.

### Implementation of quality control procedures

Quality control of robust amplification, library preparation and sequencing are of utmost importance for these complex assays. Different primers need to work under the same reaction conditions, while additional variability can be introduced by sample characteristics and sequencing. Primer performance must be monitored longitudinally, and for the exact estimation of clonal abundance it is important to correct for the number of sequencing reads per input molecule.

To address these issues, we established and validated two types of quality control procedures: (i) a ‘central in-tube quality control’ (cIT-QC) spiked to each tube as library control and calibrator, and (ii) a ‘central poly-target quality control’ (cPT-QC), or run control, to monitor general primer performance and sequencing.

To compose the cIT-QC, IG/TR rearrangements of many human lymphoid cell lines were comprehensively characterized by amplicon- and capture-based NGS and Sanger sequencing. Nine cell lines were selected to form the cIT-QC with at least three different clonal rearrangements for each of the eight PCR tubes, totalling 24 rearrangements. The current design requires an equal number of cell line DNA copies to be spiked into each tube, as described below.

For the cPT-QC a mixture of different lymphoid specimens was considered to cover the whole IG/TR repertoire more comprehensively. To this end we produced material consisting of equal ratios of DNA from peripheral blood mononuclear cells (MNCs), thymus and tonsil. For more details see accompanying manuscript by Knecht et al. [30].

### Laboratory protocol

Primers were tailed with universal and T7-linker sequences, and divided over eight tubes (IGH-VJ, IGH-DJ, IGK-VJ-Kde, intron-Kde, TRB-VJ, TRB-DJ, TRG, TRD). The PCR protocol is summarized in Table 1. Sequencing libraries were prepared via a two-step PCR, each using a final reaction volume of 50 µl with 100 ng diagnostic DNA and 10 ng of polyclonal DNA. For the cIT-QC, 40 cell equivalents of the nine different cell lines were spiked into all samples (see accompanying manuscript by Knecht et al. [30]). MgCl_2_ was intended to be used at a final concentration of 1.5 mM, but needed optimization for some tubes. Therefore, master-mixes for the 1st PCR were tube-specific, but the temperature profile was uniform for all tubes. Concentrations of all primers are shown in Supplementary Table 3. After 1st PCR, gel electrophoresis was performed to check for successful amplification of all targets. For TRB, gel extraction of the specific PCR products was performed prior to the 2nd PCR.

**Table 1:** Standardized PCR protocol

(a) Reaction conditions of 1st and 2nd PCR													
1st PCR													
	Stock concentration	IGH V-J		IGH D-J		IGK-VJ-Kde, intron-Kde		TRB V-J, D-J		TRG		TRD	
		Final concentration	μl/library	Final concentration	μl/library	Final concentration	μl/library	Final concentration	μl/library	Final concentration	μl/library	Final concentration	μl/library
PCR Buffer II	10×	1×	5	1×	5	1×	5	1×	5	1×	5	1×	5
MgCl_2_	25 mM	2.5 mM	5	3 mM	6	1.5 mM	3	4 mM	8	4 mM	8	2 mM	4
dNTP-Mix	10 mM	0.2 mM	1	0.4 mM	2.0	0.2 mM	1	0.2 mM	1	0.2 mM	1	0.2 mM	1
EagleTaq/AmpliTaq Gold	5 U/μl	1 U/rxn	0.2	1.5 U/rxn	0.3	1 U/rxn	0.2	1 U/rxn	0.2	1 U/rxn	0.2	1 U/rxn	0.2

2nd PCR			
	Stock concentration	all tubes	
		Final concentration	μl/sample
PCR buffer with MgCl_2_	10×	1×	5
	18 mM	1.8 mM	0
dNTP-Mix	10 mM	0.2 mM	1
Fast Start High Fidelity polymerase	5 U/μl	2.5 U/rxn	0.5

(b) Cycling conditions							
1st PCR				2nd PCR			
1 cycle	Initial denaturation	94 °C	10 min	1 cycle	Initial denaturation	95 °C	2 min
35 cycles	Denaturation	94 °C	1 min	20 cycles	Denaturation	94 °C	30 s
	Annealing	63 °C	1 min		Annealing	63 °C	30 s
	Extension	72 °C	30 s		Extension	72 °C	30 s
1 cycle	Final extension	72 °C	30 min	1 cycle	Final extension	72 °C	5 min
		12 °C	∞			12 °C	∞

Reaction volume: 50 µl

All 1st round PCR products, except TRB PCR products, were diluted 1:50 unless amplicons were very weak. TRB PCR products and PCR products with weak amplicons were used undiluted. Master-mixes for the 2nd PCR and the temperature profiles were identical for all tubes (Table 1). Primers for the 2nd PCR contained sequencing adaptors and sequencing indexes (barcodes). Unique combination of forward and reverse indexes was used for each library. Three microlitres of undiluted TRB PCR products and 1 µl of 1:50-diluted IGH, IGK, TRG and TRD PCR products were amplified in the 2nd PCR.

Following 2nd PCR, products from all samples of a run were pooled in equimolar ratios into eight tube-wise subpools and purified by gel extraction (see Table 2 for the amplicon lengths). Finally, the subpools were pooled equimolarly into one final pool. Sequencing was performed on Illumina MiSeq sequencers, using 2 × 250 bp v2 chemistry with a final concentration of 7 pM for the amplicon library and 10% PhiX control added to avoid low-complexity library issues. The detailed standard operating procedure is provided as supplementary information.

**Table 2:** Mean size of PCR products after the 2nd PCR (containing the Illumina sequencing adaptors and barcodes)

Gene	Amplicon length (bp)
TRB-VJ	309–407
TRB-DJ	300–408
TRG	256–360
TRD	309–450
IGH-VJ	484–681
IGH-DJ	266–358
IGK-VJ-Kde	296–384
intron-Kde	309–382

### Bioinformatic protocol

ARResT/Interrogate [32] was the main bioinformatics platform used in this study. Both Vidjil [37] and IMGT [38] resources are available through ARResT/Interrogate as built-in tools and were employed for specific aspects of this work, mainly analysis of rearrangements with unclear annotation. Data are deposited at EMBL/EBI European Nucleotide Archive (ENA), accession code PRJEB32668.

Demultiplexing was performed accepting no mismatches. Reads were annotated with EuroClonality-NGS primer sequences (to trim non-amplicon sequences, and for the cPT-QC-based quality control), paired-end joined, dereplicated, immunogenetically annotated [39], and eventually classified into rearrangement types (complete and incomplete, and other special types like intron-Kde rearrangements), or ‘junction classes’. Reads without rearrangement were excluded from the total read count used for relative abundances.

cIT-QC sequences described above and elsewhere (see accompanying manuscript by Knecht et al. [30]), were identified in the data through their immunogenetic annotation. Their counts served both as ‘in-tube’ control and for normalization per primer set: total cIT-QC cells are divided by cIT-QC total reads, the resulting factor used to convert rearrangement reads to cells, and those cells then further divided by total input cells (15,000 in this study). Identified IG/TR sequences were defined as index sequences if their abundance after cIT-QC normalisation exceeded 5%.

ARResT/Interrogate can track the DNJ 3′ stem of a junction, the sequence remaining stable during IGH or TRB clonal evolution in case of V replacement or ongoing V to DJ rearrangements. The stem consists of the last ≤ 3nt of D (or of the NDN if no D is identifiable), any and all of N2 nucleotides, and the J nucleotides of the junction. This stem is available as a separate immunogenetic feature across all samples and thus can be linked to other features, e.g. clonotypes.

### Multicentre validation of assays for MRD marker identification in ALL

**Fig. 2:**
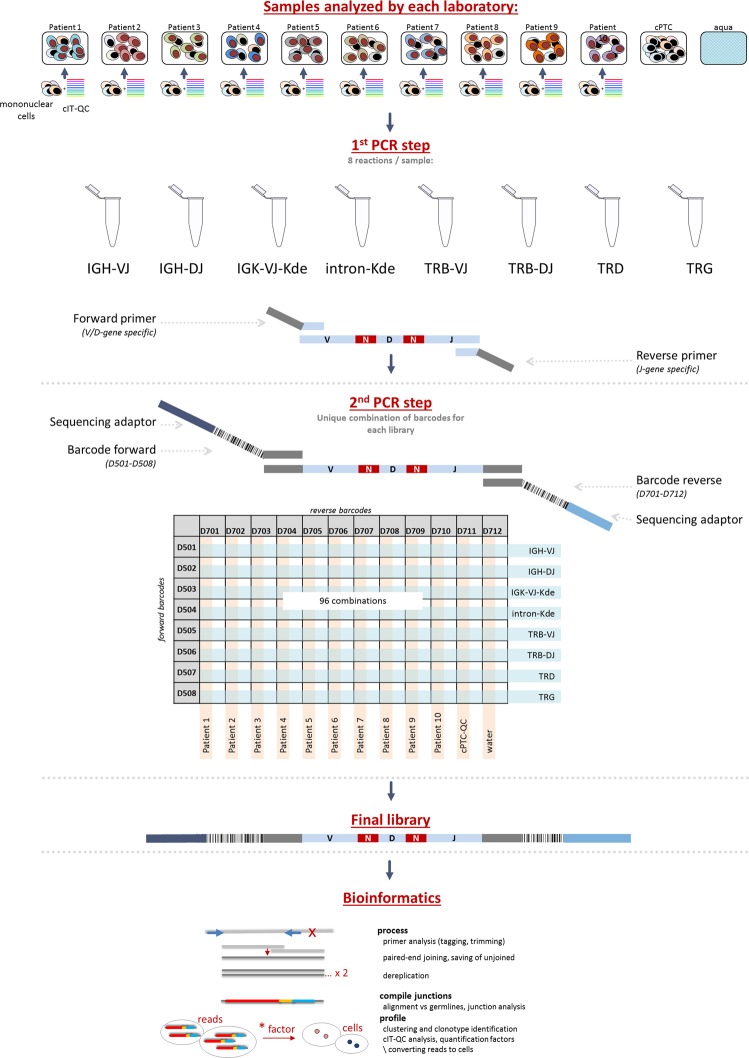
Schematic overview of the workflow for multicentre validation of IG/TR NGS assays for MRD marker identification in ALL. The IG and TR gene rearrangements are amplified in a two-step approach using multiplex PCR assays. Each of the participating laboratories performed NGS-based IG/TR MRD marker identification in 10 patients with ALL. A central polytarget control (cPT-QC) was used to monitor primer performance, and central in-tube controls (cIT-QC) were spiked to each sample as library-specific quality control and calibrator. Pipetting was performed in a 96-well format. The data analysis was performed using ARResT/Interrogate

Next, 50 ALL diagnostic samples (29 BCP-ALL and 21 T-ALL; Supplementary Table 4) were analysed for the multicentre validation study. Each of the five participating laboratories received preconfigured 96-well plates containing the different multiplexed NGS primer combinations per target (Fig. 2).

In total, 96 libraries were generated per lab (total of 480 libraries), and sequenced with a collective output of 47M reads (⌀ 9.2 M/lab). Centralised analysis was performed with ARResT/Interrogate [32] using IMGT germline sequences [39]—further analyses and verifications were performed with Vidjil [37] and IMGT/V-QUEST [38].

**Fig. 3:**
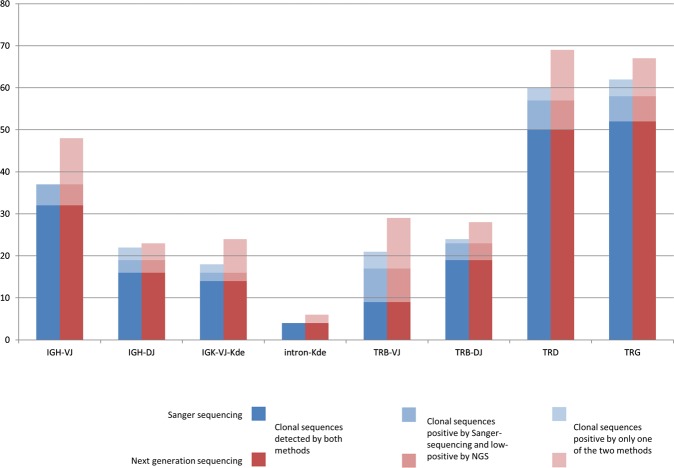
Results of multicentre validation of assays for MRD marker identification in ALL. Blue: Index sequences identified by Sanger sequencing. Red: Index sequences identified by NGS. Darkest blue/red are clonal sequences identified by both methods; lightest blue/red are sequences identified only by the respective method. Median blue/red are clonal sequences identified by both methods, but by NGS with an abundance of <5% after normalization

Overall, 311 clonal IG/TR rearrangements (clonotypes) were identified, with a mean of 5.2 (0–14)/sample by NGS (a 5% threshold was applied for NGS after cIT-QC-based normalization) vs. 5.0 (0–14)/sample by Sanger, while 217 (45%) libraries demonstrated no clonotypes above threshold by either method. A total of 196/311 (63%) clonotypes were fully concordant between NGS and Sanger (Fig. 3). NGS exclusively identified 63/311 (20%) index sequences, whereas 52/311 (17%) IG/TR Sanger sequences were not assigned as NGS index sequence by ARResT/Interrogate. 26/63 NGS positive/Sanger negative cases showed a clonal PCR product also in the respective low-throughput approach but subsequent Sanger sequencing failed due to polyclonal background, mixed sequences or weak PCR products. In an additional 6/63 NGS positive/Sanger negative cases the respective primer was missing in the low-throughput approach. For the remaining 31/63 discrepancies no technical explanation for Sanger failure could be found. In 16/19 q/ddPCR evaluated cases the rearrangement was confirmed by ASO-PCR, in three of these on a subclonal level.

Conversely, 52/311 clonal IG/TR rearrangements were detected by Sanger sequencing only, when applying the 5% NGS threshold: for 5/52 sequences (1 TRG, 2 TRB-VJ and 2 IGH-DJ) the relevant primer was not present in the NGS primer set, in 12/52 cases no explanation was found for the discrepancy. However, in most discordant cases (35/52) the Sanger identified sequences (7 TRD, 8 TRB-VJ, 6 TRG, 4 TRB-DJ, 2 IGK-VJ-Kde, 5 IGH-VJ and 3 IGH-DJ) that were also detectable by NGS, but with an abundance below 5%. In 36/39 q/ddPCR evaluated cases the rearrangement was confirmed by ASO-PCR (including all low NGS positive sequences), in 14 of these on a subclonal level. The overall concordance between Sanger and NGS, including negative libraries, was 78%.

**Fig. 4:**
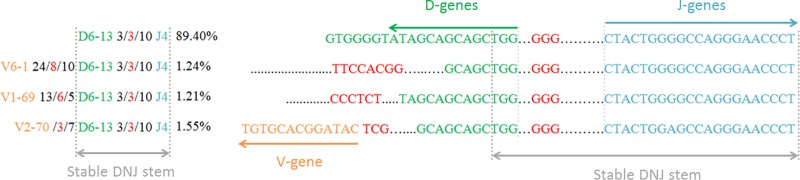
Clonal evolution in a BCP-ALL patient. The dominant incomplete IGH rearrangement (IGHD6-13 - IGHJ4) was identified with an abundance of 89.4% together with three additional complete IGH rearrangements with lower abundance (1.21–1.55%) and the same DNJ sequence. Only the CDR3 region is shown for each sequence

Interestingly, in 12/29 B-lineage ALL samples the evolution of the dominant clonal IGH sequence was identified employing a special tool in ARResT/Interrogate. The evolved clonotypes shared the DNJ stem with the dominant one, but the VND part of the rearrangement differed (example in Fig. 4).

Assay performance was also analysed by standardized evaluation of QC samples (cIT-QC and cPT-QC, see accompanying manuscript by Knecht et al. [30]) and showed high intra- and inter-lab consistency without statistically significant differences between the five labs.

### Modifications of the central SOP

During the process of multicentre validation, modifications of the SOP were tested in particular laboratories as parallel projects.

#### One-step versus two-step PCR

It was decided to use two-step PCR to enable switching of sequencing adaptors and to limit the total number of required primer batches even if a large number of barcodes is required. As first round PCR products are not barcoded, identification of contamination phenomena is hampered in this approach. Therefore, a one-step PCR was tested in a single centre (Paris). The one-step approach reduces the risk of contamination and thus favours use of NGS not only for marker identification, but also for MRD assessment. The standard operating procedure is shown in Supplementary information.

#### Use of Ion Torrent platform

Ion Torrent platform was tested in a single-centre setting (Prague) and showed a very good concordance (*R*^2^ = 0.89) with the standard approach. The standard operating procedure is shown in Supplementary information.

#### Removal of polyclonal DNA from reaction mix

Polyclonal DNA was added to each reaction in order to prevent excessive primer dimer formation in samples lacking particular rearrangements. The addition of polyclonal DNA, however, alters the composition of polyclonal background of the samples and hampers the analysis of the immune repertoire. We therefore performed testing on four samples with B- and four samples with T-cell aplasia and showed that addition of cIT-QC is sufficient to prevent the excessive formation of unspecific PCR products (see Supplementary information).

#### Bead extraction

During the single target evaluation and validation phase, gel extraction of the specific TRB amplicons turned out to lead to more specific libraries compared with bead extraction. However, gel extraction is not used in all laboratories, therefore, in a later phase of the study bead purification of all libraries was also tested. Optimization of the purification processes led to comparable ratios of specific reads irrespective of the type of library purification (Supplementary Table 5).

## Discussion

Amplicon-based IG/TR NGS provides an elegant method to detect clonality, identify MRD markers and monitor MRD in lymphoid malignancies. However, comprehensive SOPs for all relevant IG/TR targets, applicable QC procedures, suitable bioinformatic tools, and validation of the technology in a scientifically controlled, multicentre setting are still lacking [19].

Here we describe an in vitro and in silico protocol for the diagnostic identification of IG/TR MRD markers in ALL, and demonstrate its robustness and applicability across five European laboratories. EuroClonality-NGS primer sets were successfully used with high reproducibility and good concordance to Sanger sequencing, identifying on average 4% more markers per patient than classical low-throughput methods. NGS was particularly successful in correctly identifying bi-allelic rearrangements, which are technically challenging for Sanger sequencing because this requires prior separation of the respective clonal PCR products. NGS also performs better in the presence of a background of polyclonal rearrangements. Besides, it allows a more comprehensive coverage of rearrangement types. The EuroClonality-NGS TRD assay for example not only detects all types of complete and incomplete TRD gene rearrangements but also VD-JA29 recombinations [40], present in about 20% of all B-cell precursor (BCP) ALLs. In our current series, these TRDV2-JA29 rearrangements were detected in 7/29 BCP-ALL patients (24%), providing an attractive target for MRD monitoring. Notably, rearrangement coverage is not complete. The IGH-DJ tube lacks an IGHD7 primer because that would predominantly amplify the germline-configured IGH-IGHD7-IGHJ1.

Low-throughput sequencing of clonal IG/TR gene rearrangements is often cumbersome. This particularly holds true for TRB, where Sanger sequencing of clonal TRB BIOMED-2 amplicons requires a multistep approach: first with the complete set of primers to identify the rearranged genes, and second, a repetition of the sequencing reaction with gene-specific primers. In contrast, the EuroClonality-NGS assays do not require specific workflows for particular targets, thus enormously streamlining the process of MRD marker identification. This becomes increasingly important in times of MRD-based treatment requiring early patient assignment to the respective MRD risk group.

Critically, our assays provide ways to evaluate primer performance and overall quality of the whole NGS run (primers in the cPT-QC) and of each tube (spike-ins in the cIT-QC, see accompanying manuscript by Knecht et al. [30]). Such functionalities are embedded in the ARResT/Interrogate pipeline, further standardizing the whole workflow. A challenge for correct MRD marker identification in NGS data is the phenomenon of accompanying lymphoid clones that might be mixed up with the leukaemia-specific ones. Therefore, information regarding blast infiltration of the analysed sample must be related to the combined abundance information of the clonal rearrangement, the polyclonal background and the cIT-QC sequences. The integration of all this information allows for a more specific assignment compared with published approaches that define an index sequence simply as sequence with an abundance of >5% [16]. This is particularly necessary for tubes that exclusively cover rearrangements being present only in a minority of lymphoid cells (especially the TRD and intron-Kde tubes). TRD genes are not rearranged in normal B cells and are deleted in most TRγδ cells [41]. Therefore, oligoclonal TCRγδ T cells might give rise to dominant clonotypes in TRD NGS assay, in particular as the normal TCRγδ T-cell repertoire is strikingly skewed during childhood. Here the cIT-QC-based abundance correction is of utmost importance to avoid miss-assignment of (minor) clonal TRD rearrangements from minor TCRγδ cell populations as leukaemic rearrangements. Also, knowledge on rearrangement patterns in ALL is important. BCP-ALL features neither complete TRD, nor TRBJ1 gene rearrangements, T-ALL in contrast generally does not harbour complete IGH or IGK gene rearrangements [42]. Hence, identification of such rearrangements would actually reflect more the presence of accompanying T- and B-cell clones, respectively. This immunogenetic knowledge is of particular importance if marker identification is performed, e.g. at relapse after stem cell transplantation, when patients often harbour a restricted B- and T-cell repertoire. The EuroClonality-NGS approach allows for the bioinformatic identification and correction of this phenomenon, whereas conventional low-throughput approaches do not harbour correction mechanisms. Nevertheless, we urge caution in assignment of minor clones to the ALL. Although smaller subclones might be missed based on an abundance threshold (which largely explains discrepancies between Sanger sequencing and NGS in our study), decreasing the threshold would be at the expense of specificity.

Oligoclonality is a well-known phenomenon in ALL that hampers conventional IG/TR MRD [43] assessment, but this can be better identified by NGS. Multiple IG/TR gene rearrangements in ALL result from both continuing rearrangement processes (e.g. continuing IGHV to DJ joining) and from secondary rearrangements (e.g. IGH-DJ replacements, V replacement in a complete IGH rearrangement) [23, 44–49]. In 12 of 29 (41.4%) patients with B-lineage ALL, a dominant clonal IGH rearrangement was subjected to clonal evolution, resulting in the presence of smaller subclones with the same D-J stem, but different V-genes. D-J replacements are also an evolutionary possibility but cannot be unambiguously discriminated from unrelated lymphoid clones even with sophisticated bioinformatic tools.

Modifications to the here described EuroClonality-NGS assays would be possible, and have actually been tested and approved to be suitable within the working group. In particular, a one-step instead of the two-step PCR presented here might be a reasonable alternative for sites that would apply NGS not only for marker identification but also for MRD assessment. Finally, the Ion Torrent platform was successfully tested as a replacement for the Illumina MiSeq used in this study, and has subsequently also been applied more extensively for clonality assessment in formalin-fixed paraffin-embedded tissue (see accompanying manuscript by Scheijen et al. [31]).

In summary, the EuroClonality-NGS developed an IG/TR marker identification protocol, which was validated across many expert European laboratories. It covers in vitro and in silico requirements and allows for quality-controlled, streamlined, comprehensive detection of clonal IG/TR rearrangements in ALL. Compared with low-throughput methods, more MRD markers are identified, sensitivity is increased, processing time is reduced and labour-intensive conventional methods to resolve mixed sequences in case of bi-allelic rearrangements or background are avoided. In parallel, the ARResT/Interrogate bioinformatic platform has been developed with specific functionalities addressing potential pitfalls of IG/TR marker identification in ALL, thus enabling a standardized workflow. In addition, the presented approach forms the basis for future applications in clonality assessment, repertoire analysis and MRD quantification in a quality-controlled and accreditable assay with the potential to meet the upcoming European criteria (EU Regulation 2017/746) for in vitro diagnostics.

## Supplementary information

Supplementary Figures and Tables

Supplementary Information
