# Supplementary material for: Standardized next-generation sequencing of immunoglobulin and T-cell receptor gene recombinations for MRD marker identification in acute lymphoblastic leukaemia; a EuroClonality-NGS validation study
Source: Leukemia. 2019 Jun 26;33(9):2241–53. doi: 10.1038/s41375-019-0496-7 (PMC6756028; doi:10.1038/s41375-019-0496-7)
Supplement: Supplementary file 1 — Supplementary Figures and Tables [file 41375_2019_496_MOESM1_ESM.docx]

**Supplementary Figures and Tables to:**

**Standardized next-generation sequencing of immunoglobulin and T-cell receptor gene recombinations for MRD marker identification in acute lymphoblastic leukemia; a EuroClonality-NGS validation study**

Monika Brüggemann^1*^, Michaela Kotrová^1,2*,^ Henrik Knecht^1^, Jack Bartram^3^, Myriam Boudjogrha^4^, Vojtech Bystry^5^, Grazia Fazio^6^, Eva Froňková^2^, Mathieu Giraud^7^, Andrea Grioni^6^, Jeremy Hancock^8^, Dietrich Herrmann^1^, Cristina Jiménez^9^, Adam Krejci^5^, John Moppett^10^, Tomas Reigl^5^, Mikael Salson^7^, Blanca Scheijen^11^, Martin Schwarz^1^, Simona Songia^6^, Michael Svaton^2^, Jacques JM van Dongen^12^, Patrick Villarese^13^, Stephanie Wakeman^8^, Gary Wright^3^, Giovanni Cazzaniga^6^, Frédéric Davi^4^, Ramón García-Sanz^9^, David Gonzalez^14^, Patricia JTA Groenen^11^, Michael Hummel^15^, Elizabeth A. Macintyre^13^, Kostas Stamatopoulos^16^, Christiane Pott^1^, Jan Trka^2^, Nikos Darzentas^1,5^, Anton W. Langerak^17^, on behalf of the EuroClonality-NGS working group

# Supplementary Figure 1: Schematic diagram of the working group structure.


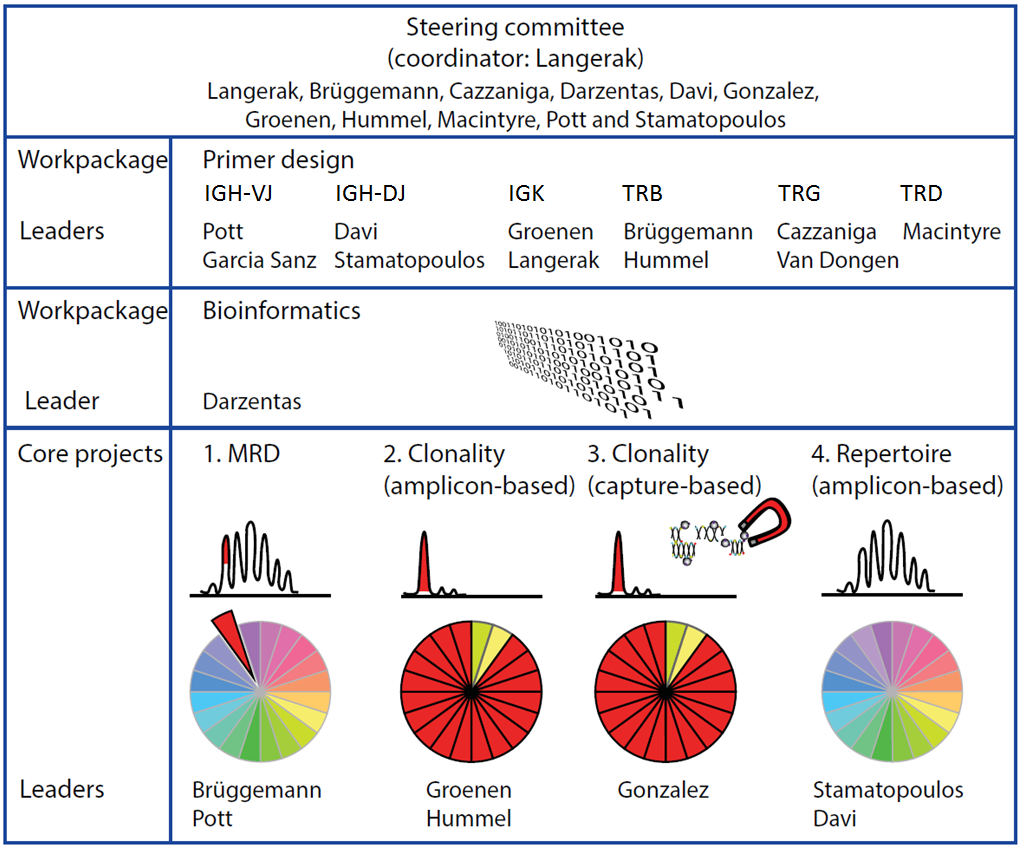


**Supplementary figure 2: Junction nucleotide length histograms**

2A) Histograms showing junction nucleotide lengths of complete IGH rearrangements (IGH-VJ tube) in a BCP-ALL patient, cPT-QC, BC, thymus, and tonsil. Bars are coloured according to the V-J genes combination.

2B) Histograms showing junction nucleotide lengths of incomplete IGH rearrangements (IGH-DJ tube) in a BCP-ALL patient, cPT-QC, BC, thymus, and tonsil. Bars are coloured according to the D-J genes combination.

2C) Histograms showing junction nucleotide lengths of IGK-VJ and IGK-V-Kde rearrangements (IGK-VJ-Kde tube) in a B-ALL patient, cPT-QC, BC, thymus, and tonsil. Bars are coloured according to the V-J-Kde genes combination.

2D) Histograms showing junction nucleotide lengths of intron-Kde rearrangements (intron-Kde tube) in a BCP-ALL patient, cPT-QC, BC, thymus, and tonsil.

2E) Histograms showing junction nucleotide lengths of complete TRB rearrangements (TRB-VJ tube) in a T-ALL patient, cPT-QC, BC, thymus, and tonsil. Bars are coloured according to the V-J genes combination.

2F) Histograms showing junction nucleotide lengths of incomplete TRB rearrangements (TRB-DJ tube) in a T-ALL patient, cPT-QC, BC, thymus, and tonsil. Bars are coloured according to the D-J genes combination.

2G) Histograms showing junction nucleotide lengths of TRG rearrangements (TRG tube) in a T-ALL patient, cPT-QC, BC, thymus, and tonsil. Bars are coloured according to the V-J genes combination.

2H) Histograms showing junction nucleotide lengths of TRD rearrangements (TRD tube) in a T-ALL patient, cPT-QC, BC, thymus, and tonsil. Bars are coloured according to the V-D-J genes combination.

**Supplementary figure 2A**

**
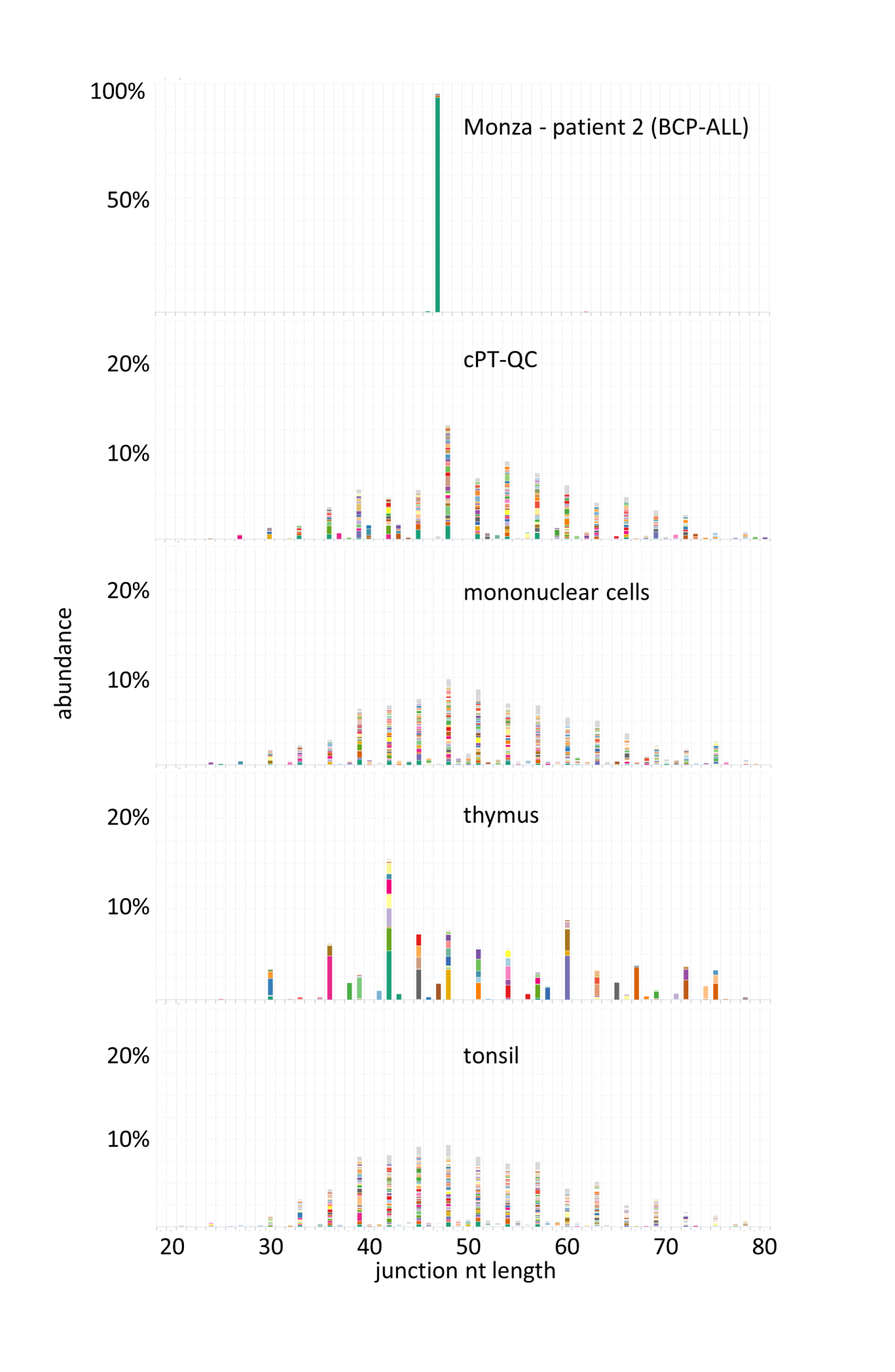
**

**Supplementary figure 2B**

**
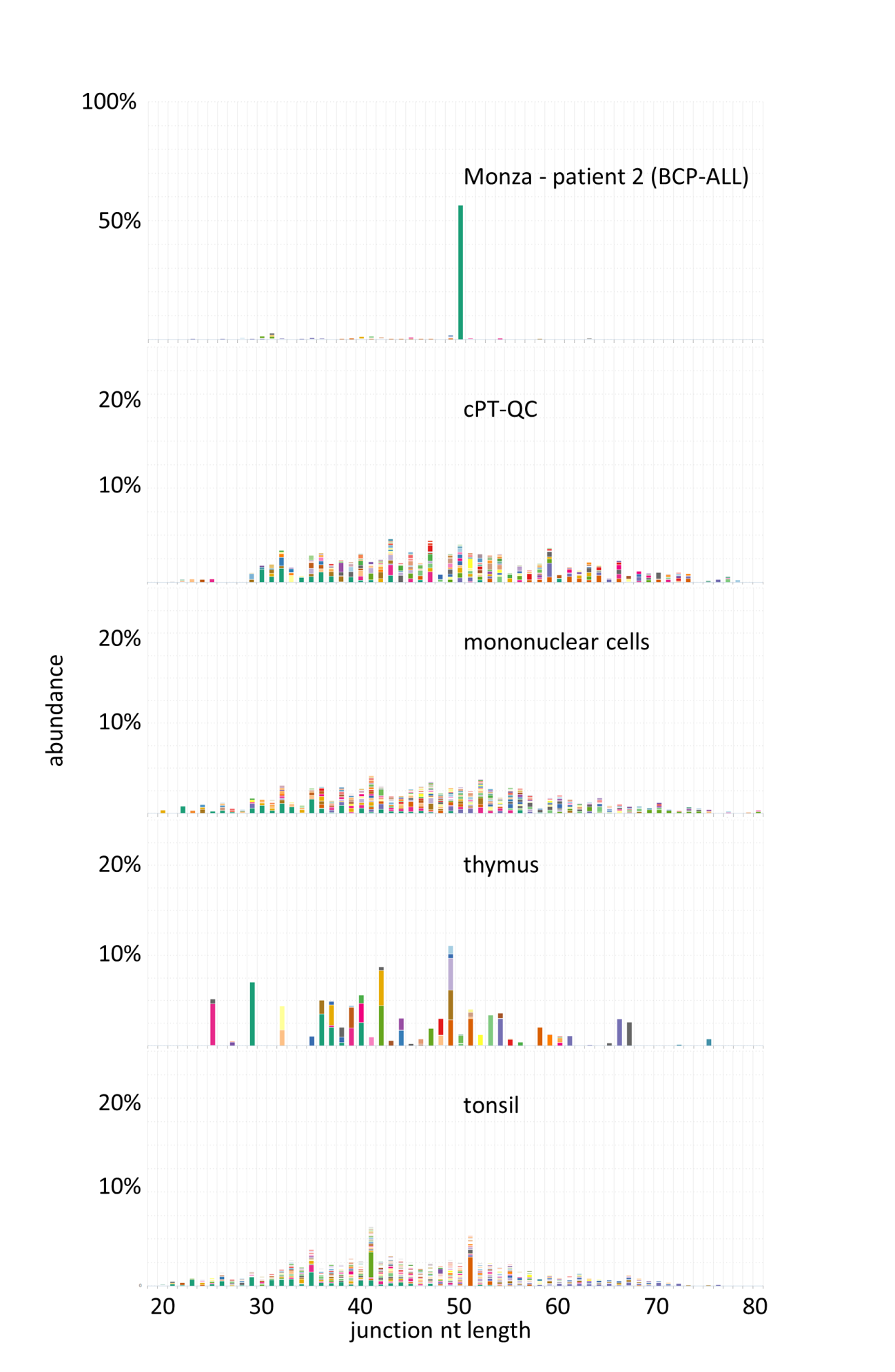
**

**Supplementary figure 2C**

**
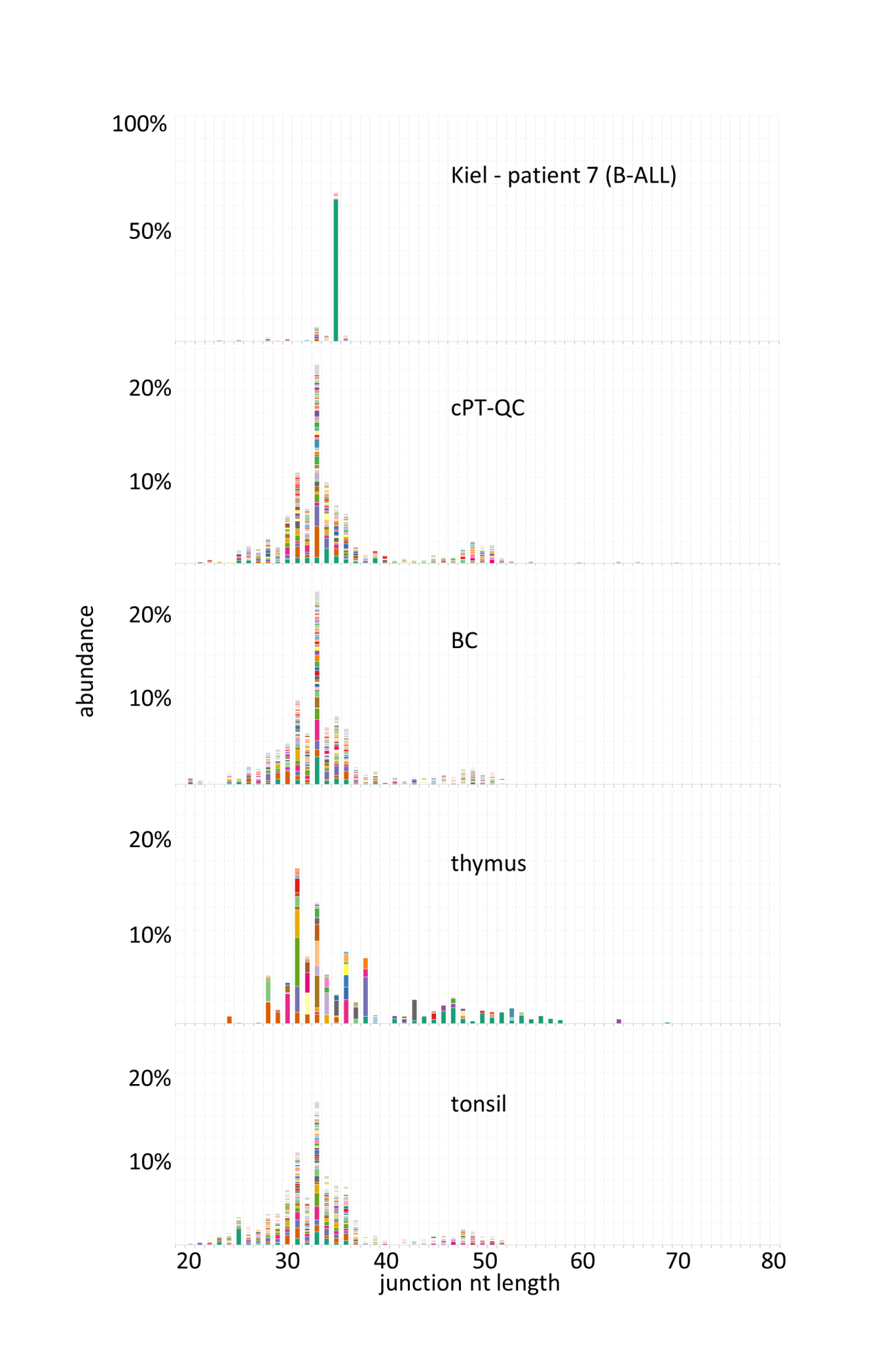
**

**Supplementary figure 2D
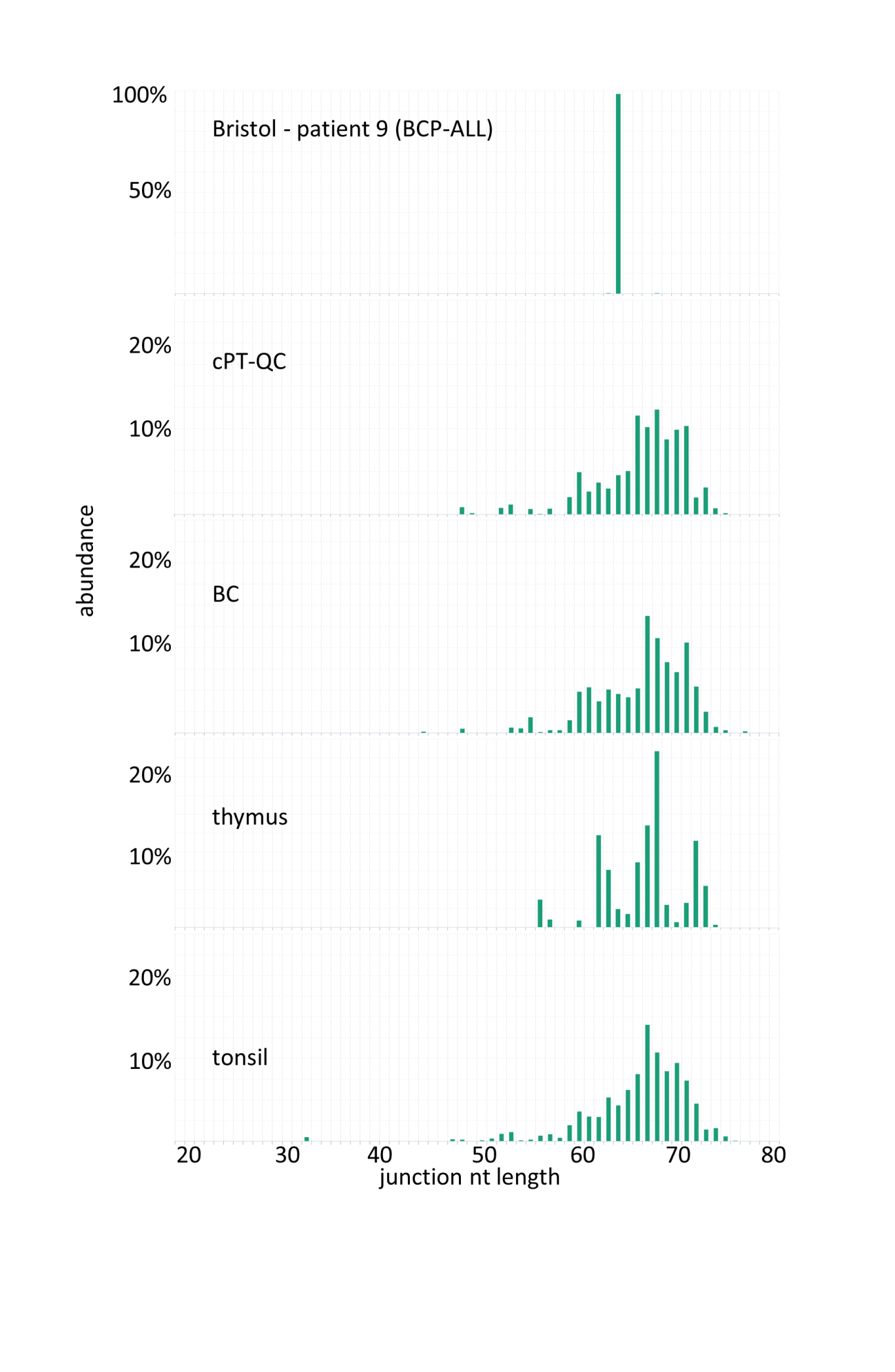
**

**Supplementary figure 2E**

**
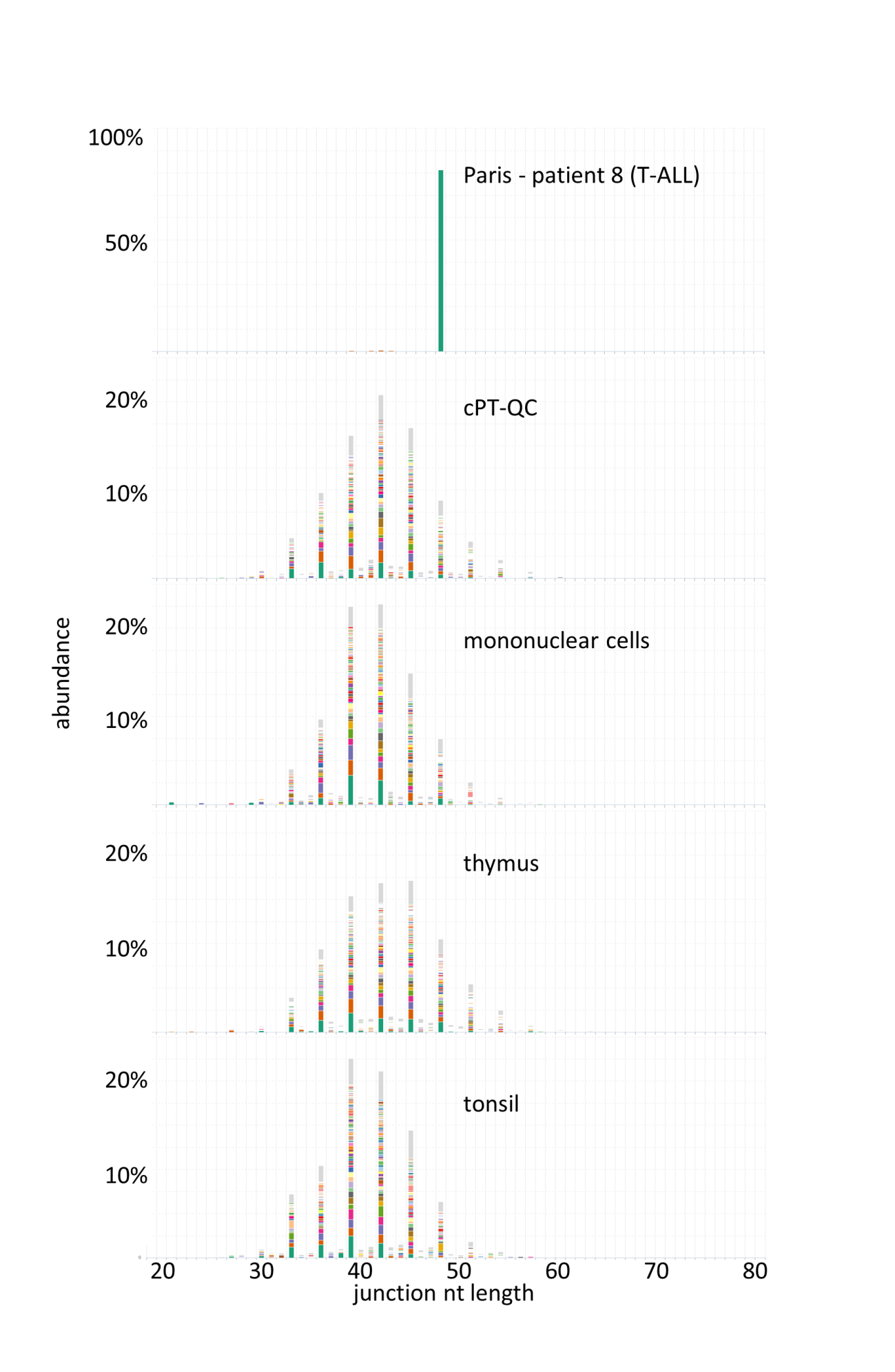
**

**Supplementary figure 2F**

**
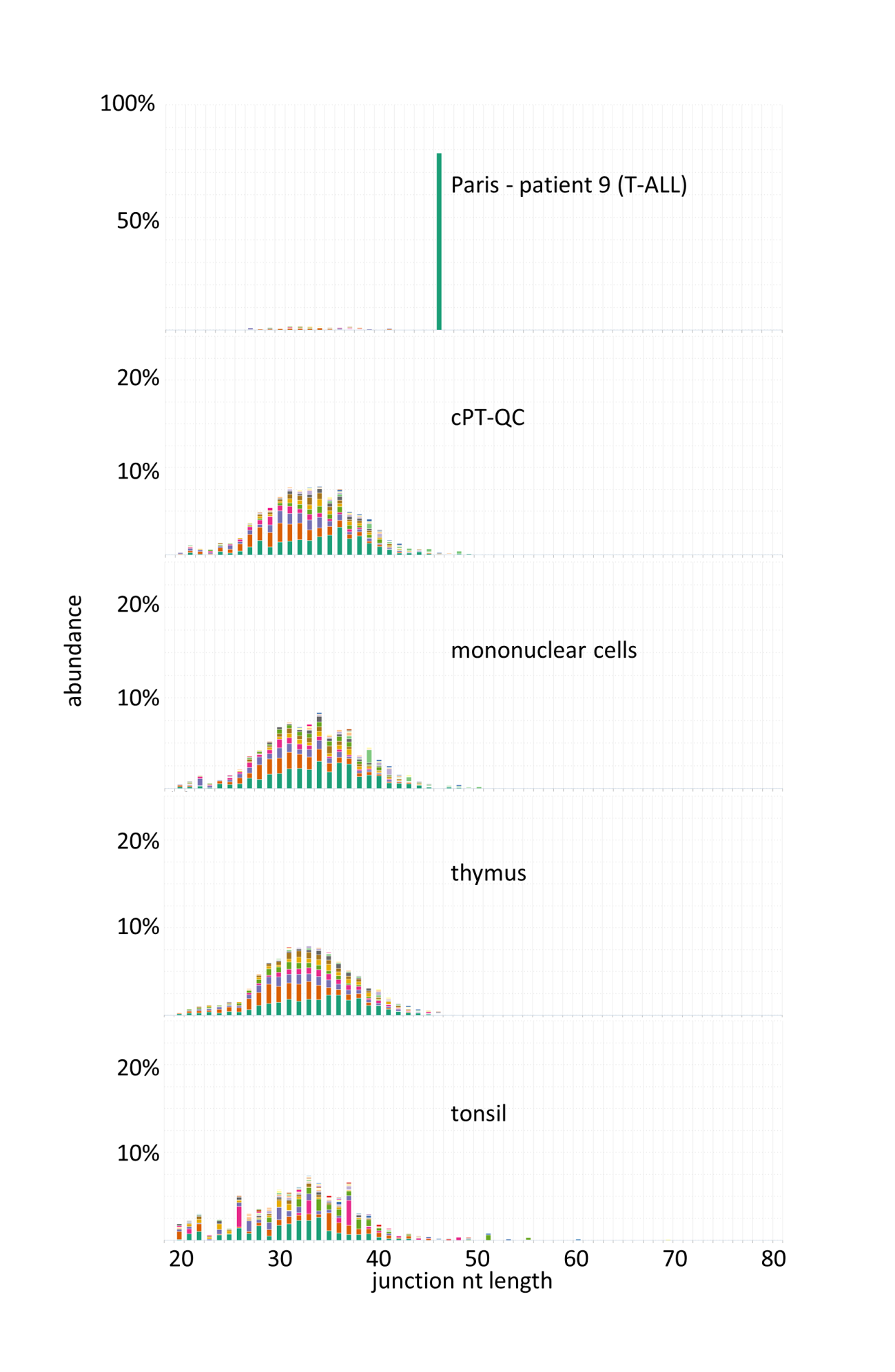
**

**Supplementary figure 2G**

**
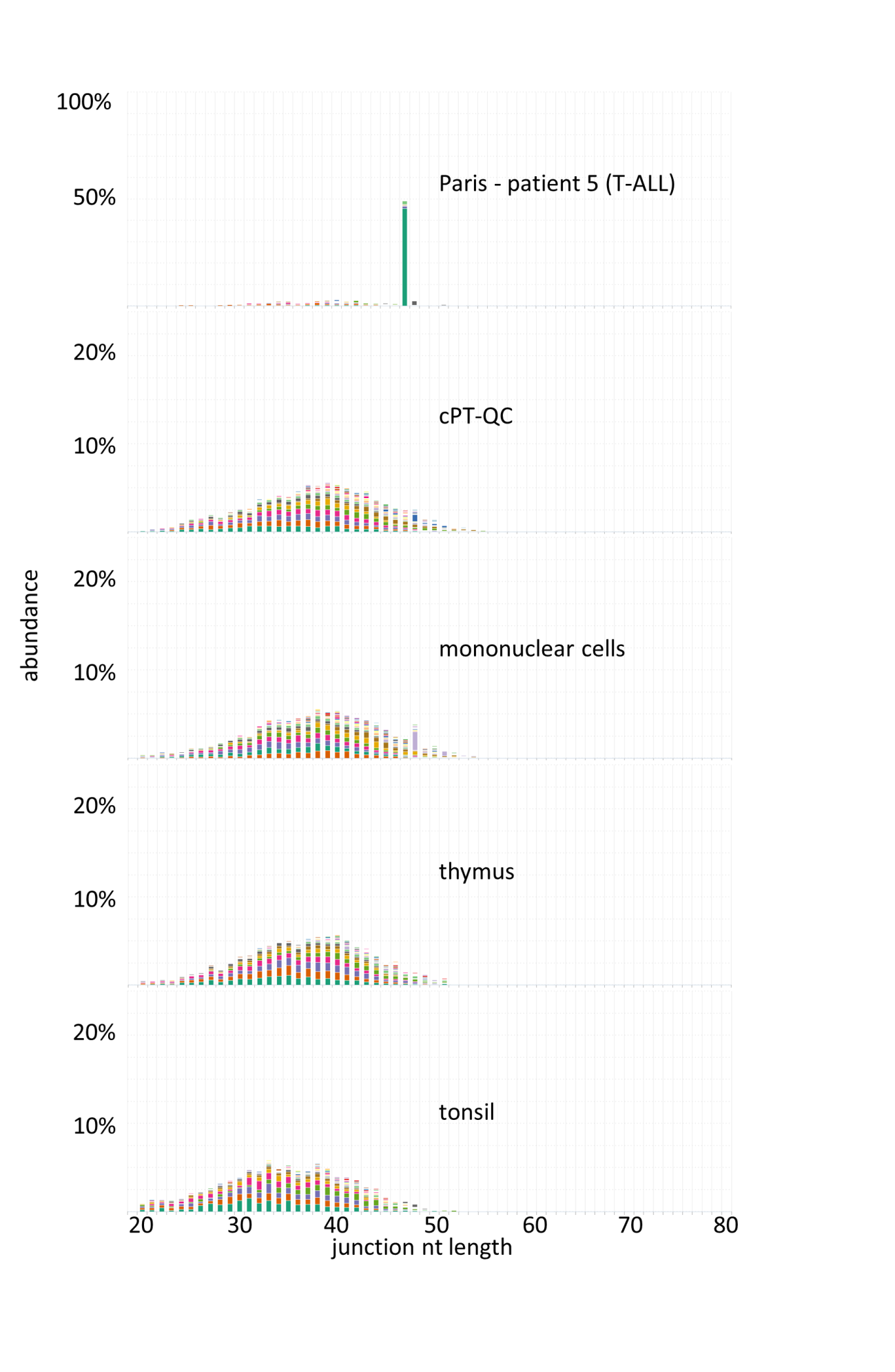
**

**Supplementary figure 2H**

**
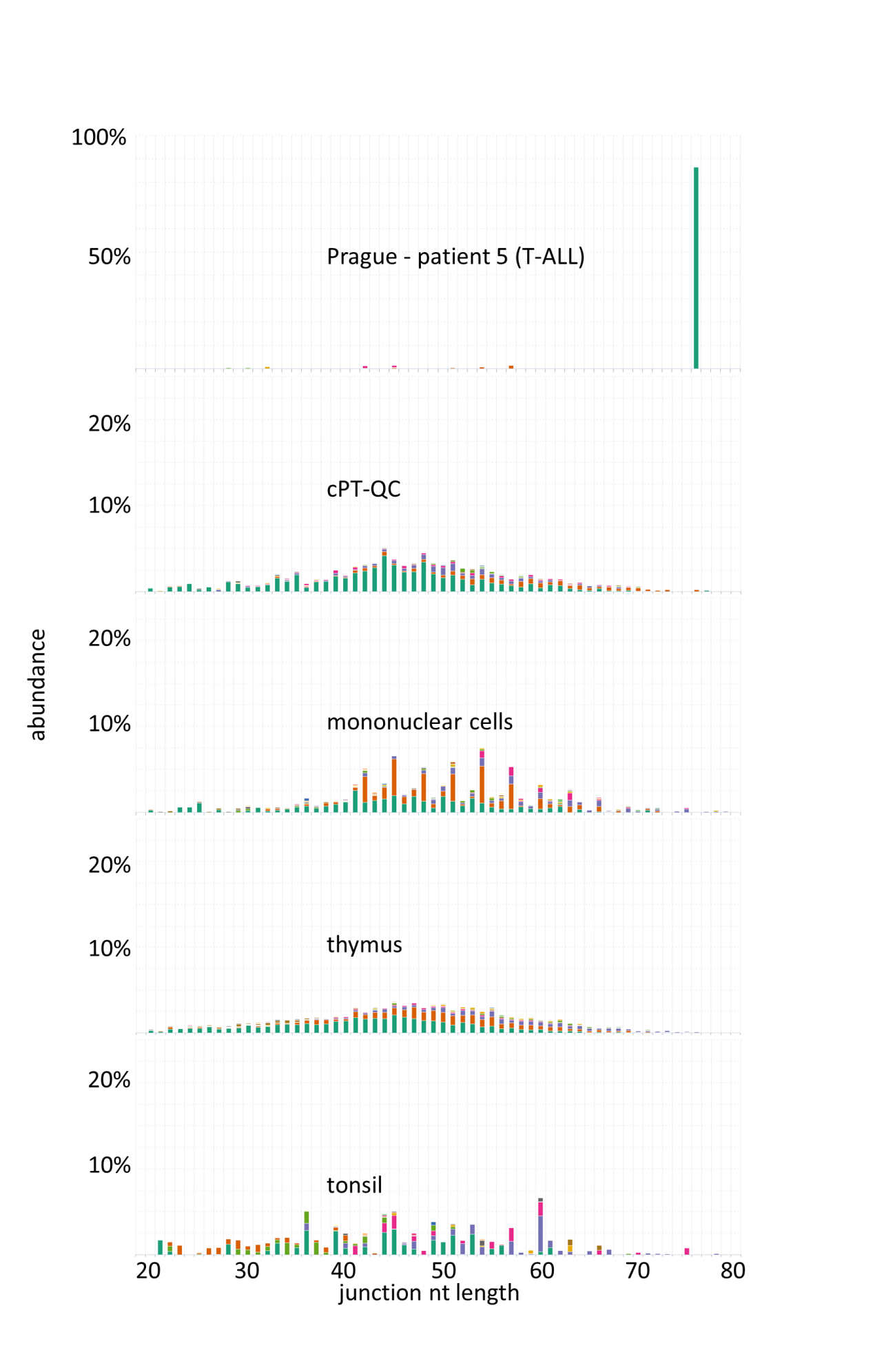
**

# Supplementary Table 1: Meetings of the EuroClonality-NGS working group

| **No.** | **Year** | **Date** | **Location** | **Aim/topics** |
| --- | --- | --- | --- | --- |
| 1 | 2012 | May 10-11 | Hoofddorp, the Netherlands | Kick-off meeting |
| 2 | 2012 | September 17-18 | Berlin, Germany | General meeting |
| 3 | 2012 | November 26-27 | Rotterdam, the Netherlands | Primer design and bioinformatics meeting |
| 4 | 2013 | January 28-29 | Rotterdam, the Netherlands | Primer design and bioinformatics meeting |
| 5 | 2013 | June 6-7 | Paris, France | Primer design and bioinformatics meeting |
| 6 | 2013 | November 6-8 | Rotterdam, the Netherlands | General meeting |
| 7 | 2014 | March 3-4 | Chania / Crete, Greece | General meeting |
| 8 | 2014 | Sep 29-Oct 1 | Rotterdam, the Netherlands | General meeting |
| 9 | 2015 | February 18-20 | Prague, Czech Republic | General meeting |
| 10 | 2015 | October 5-6 | Rotterdam, the Netherlands | General meeting |
| 11 | 2016 | April 13-15 | Kiel, Germany | General meeting |
| 12 | 2016 | October 3-5 | Thessaloniki, Greece | General meeting |
| 13 | 2017 | April 3-5 | Uppsala, Sweden | General meeting |
| 14 | 2017 | October 11-13 | Belfast, Northern Ireland | General meeting |
| 15 | 2018 | April 9-11 | Lille, France | General meeting |

Supplementary Table 2: **Overview over EuroClonality-NGS primer sets testing and validation rounds**

**Supplementary Table 2A: Design of the IGH-VJ-FR1 panel in consecutive testing rounds**

| **Version** |  | **Time frame** | **Number of FPs** | **Number of RPs** | **Tested reaction conditions** | **Tested clonal controls** | **Tested polyclonal controls** | **Tested neg controls** | **Involved labs** | **Consequence** |
| --- | --- | --- | --- | --- | --- | --- | --- | --- | --- | --- |
| 1 | Initial design | Sept 2012-Oct 2013 | 26 | 4 | Primer concentrations: 0.025/0.05/0.1/0.2 µM each  MgCl2: 1.5/3/4/5 mM  Annealing temperature: 54/57/60/63°C  Cycle numbers:  1^st^ PCR: 20/25/30/35 cycles  2^nd^ PCR: 20/25/30/35 | Ramos cell line | PBMNCs | Aqua  HeLa cell line | Kiel | Exclusion of 16 V-primers covering pseudo genes and the redundant ones |
| 2 | Multicentre validation (Pilot1) | January – February 2014 | 10 | 4 | Primer concentrations:0.1 µM each,  MgCl2: 1.5mM  Annealing temperature: 63°C  Cycle numbers:  1^st^ PCR: 35 cycles  2^nd^ PCR: 20 cycles | Spike-in (CA-46 + GRANTA + TOM-1)  37 patient samples pool with 39 rearranged IGH | Mixed MNCs 20 healthy donors | HeLa cell line  Aqua | Kiel  Torino  Monza  London  Prague |  |
| 3 | Single-centre validation | 03-10/2014 | 10 | 4 | Primer concentrations: individualized within multiple testing rounds  MgCl2: 1.5mM  Annealing temperature: 63°C  Cycle numbers:  1^st^ PCR: 35 cycles  2^nd^ PCR: 20 cycles | 26 B-cell lines pool (29 rearrangements)  35 patient samples pool (37 rearrangements) |  | Aqua | Kiel | Addition of 5 V-primers and exclusion of 2 J-primers |
| 4 | Multicentre validation (ALL marker identification) | Spring 2016-October 2017 | 15 | 2 | Primer concentrations: adapted to 0.1/0.2 µM each,  MgCl2: 2.5mM  Annealing temperature: 63°C  Cycle numbers:  1^st^ PCR: 35 cycles  2^nd^ PCR: 20 cycles | 50 diagnostic ALL samples  Spike-in pool (9 cell lines) | Central polyclonal control pool (1/3 thymus, 1/3 tonsil, 1/3 PBMNC) | Aqua | Kiel  Prague  London/Bristol  Paris Necker  Monza | Approval of final assay for IG/TR marker identification |

**Supplementary Table 2B: Design of the IGH-DJ panel in consecutive testing rounds**

| **Version** |  | **Time frame** | **Number of FPs** | **Number of RPs** | **Tested reaction conditions** | **Tested clonal controls** | **Tested polyclonal controls** | **Tested neg controls** | **Involved labs** | **Consequence** |
| --- | --- | --- | --- | --- | --- | --- | --- | --- | --- | --- |
| 1 | Initial design | Sept 2012-Oct 2014 | 9 | 1 | Primer concentrations: 0.5/0.1/0.2 µM each  MgCl2: 1.5/2/3/4 mM  Annealing temperature: 57/60/63°C  Polymerase : Taq Gold vs Hifi Roche  Cycle numbers:  1Step PCR : 25 or 30 or 35 cycles | 6 patients with clonal rearranged  IGH-DJ | PBMNCs  Tonsils | Aqua  HeLa cell line | Paris Pitié | Removal of IGHD7 primer  Approval of reaction conditions:  Primer concentrations: 0.1 µM each  MgCl2: 2mM  Annealing temperature: 60°C  Polymerase : Hifi Roche  Cycle numbers:  1Step PCR : 35 cycles |
| 2 | Single-centre validation | Oct 2014- October 2015 | 8 | 1 | Primer concentrations: 0.1 µM each  MgCl2: 2mM  Annealing temperature: 60°C  Cycle numbers:  1Step PCR: 35 cycles | 38 patients with clonal rearranged  IGH-DJ | MNCs 4 healthy donors | Aqua  HeLa cell line | Paris Pitié |  |
| 3 | Multicentre validation (ALL marker identification) | Spring 2016-October 2017 | 8 | 1 | Primer concentrations: 0.2 µM each (FPs), 0.4 µM each (Reverse primer)  MgCl2: 3mM  Annealing temperature: 63°C  Cycle numbers:  1^st^ PCR: 35 cycles  2^nd^ PCR: 20 cycles | 50 ALL diagnostic samples  cITC (9 cell lines pool) | Central polyclonal control (1/3 thymus, 1/3 tonsil, 1/3 PBMNC) | Aqua | Kiel  Prague  London/Bristol  Paris Necker  Monza | Approval of final assay for IG/TR marker identification |

**Supplementary Table 2C: Design of the IGK-VJ-Kde and intron-Kde** **panels in consecutive testing rounds**

| **Version** |  | **Time frame** | **Number of FPs** | **Number of RPs** | **Tested reaction conditions** | **Tested clonal controls** | **Tested polyclonal controls** | **Tested neg controls** | **Involved labs** | **Consequence** |
| --- | --- | --- | --- | --- | --- | --- | --- | --- | --- | --- |
| 1 | Initial design | September 2012  -  February 2013 | 16 V and  1 intron | 3 J  and  1 Kde | (1) IGKV-IGKJ  (2) IGKV/intron-Kde  Multiplex testing  PCR volume 25 µl  0.2U Taq  primers: 0.2 µM each  MgCl_2_: 1.5 mM  Annealing temperature 60°C  Cycles PCR: 35  GeneScanning | 5 defined monoclonal IGK-VJ-Kde samples | 3 tonsils  5 defined IGK-VJ polyclonal samples;  8 IG-Kde polyclonal samples | Aqua | Nijmegen | Redesign  IGKV 1/4/5/6/7 and intron primers, addition of 4 IGKV gene- specific primers |
| 2 | Multicentre  validation | March 2013-  March 2014 | 20 V and  1 intron | 3 J  and  1 Kde | (1) IGKV-IGKJ  (2) IGKV/intron-Kde  (3) IGKV/intron-IGKJ/Kde  -Polyclonal samples multiplex (1, 2 and 3) testing;  PCR volume 25 µl  0.2U Taq  primers: 0.2 µM each  MgCl_2_:  1.0/1.5/2.0/2.5 mM  Annealing temperature 60°C  Cycles PCR: 35  GeneScanning  -Polyclonal samples monoplex testing  - B-lineage cell line controls monoplex testing  - B-lineage cell line controls multiplex (1 and 2) testing;  PCR volume 25 µl  0.2U Taq  primers: 0.2 µM each  MgCl_2_: 1.5 mM  Annealing temperature 60°C  Cycles: 35  GeneScanning | 60 defined B-lineage cell line controls | 4 tonsils, 1 spleen, mononuclear cells |  | Rotterdam  Nijmegen | Exclusion of 7 IGKV (pseudogene specific) primers,  redesign intron primer |
| 3 | Single-centre validation | March 2014  -  March 2015 | 13V and  1 intron | 3 J  and  1 Kde | (3) IGKV/intron-IGKJ/Kde  PCR volume 25 / 50 µl  0.2U Taq  primers: 0.2 µM each  DNA input 25/50 ng,  MgCl_2_:  1.5/2.0/2.5/3.0 mM  Annealing temperature 60°C  Cycles PCR: 30 or 35  Blunt-ended adaptor ligation  NGS | Kappa artificial polyclonal control (APC) ; 9 defined B-lineage cell line controls and 3 defined monoclonal samples; composed of 23 R (based on Biomed-2/EuroClonality PCRs)  3 defined B-lineage cell line controls | 2 tonsils |  | Nijmegen | Preferred condition  PCR volume 25 ul 3.0mM MgCl_2_  DNA input 50ng of artificial polyclonal control  input cycles 30x (in context of additional cycles during library preparation)  Detection of intron-Kde rearrangement is suboptimal |
| 4. | Multicentre testing 1 | March 2015  -  March 2016 | 13V  and  1 intron | 3 J  and  1 Kde | (1) IGKV-IGKJ  (2) IGKV/intron-Kde  PCR volume 25 µl  0.2U Taq  primers: 0.2 µM each  MgCl_2_: 3.0 mM  DNA input 50 ng  Annealing temperature 60°C  Cycles PCR: 30  Blunt-ended adaptor ligation  NGS | Serial dilution 2  defined B-lineage cell line controls  (with each both IGKV-IGKJ and IGKV-Kde rearrangements)  28 diagnostic B-cell monoclonal samples | 2 tonsils |  | Nijmegen  Berlin  Rotterdam | Detection of intron-Kde rearrangement needs optimization for the clonality protocol |
| 5.* | Multicentre validation (ALL marker identification) | Spring 2016-October 2017 | 11V  and  1 intron | 3J  and  1 Kde | (1) IGKV-IGKJ/Kde  (2) intron-Kde  primers: 0.1 µM each  MgCl2: 1.5mM  Annealing temperature: 63°C  Cycle numbers:  1^st^ PCR: 35 cycles  2^nd^ PCR: 20 cycles | 50 ALL diagnostic samples  cITC (9 cell lines pool) | Central polyclonal control (1/3 thymus, 1/3 tonsil, 1/3 PBMNC) | Aqua | Kiel  Prague  London/Bristol  Paris Necker  Monza | Approval of final assay for IG/TR marker identification |
| 6.** | Multicentre testing 2 | March 2016 -  May 2017 | 13V and  1 intron | 3 J  and  1 Kde | (1) IGKV-IGKJ  (2) IGKV/intron-Kde  (3) IGKV/intron-J/Kde  (4) IGKV-IGKJ/Kde  (5) intron-Kde  (6) IGKV-Kde  Testing variables:  PCR volume 25 µl  0.2U Taq  primers: 0.2 µM each, except for intron primer 0.2/ 1 /2 µM  MgCL_2 :_ 1.5/3.0 mM  DNA input 2.5/5/10/20/40 ng  Annealing temperature 60°C  Cycles PCR: 30  Blunt-ended adaptor ligation  NGS | Serial dilution a  defined B-lineage cell line control  7 diagnostic monoclonal B-cell samples | 3 tonsils,  2 lymph nodes,  3 PBMC,  1 bone marrow |  | Nijmegen  Rotterdam | Approval of final conditions for FFPE:  a IGKV-IGKJ/Kde multiplex PCR and a second intron-Kde PCR    alternatively;  a single multiplex PCR: IGKV/intron-IGKJ/Kde  followed by separate analysis of IGKV-IGKJ and IGKV/intron-Kde rearrangements in the bioinformatic pipeline;  PCR volume 25 µl  0.2U Taq  primers: 0.2 µM each  MgCl_2_: 1.5 mM  DNA input 20-40ng  Annealing temperature 60°C  Cycles: 30x |

*Adaption of the assay for IG/TR marker identification in ALL ALL

** Optimization of the Kappa assay for short fragment sequencing for clonality detection using Ion Torrent sequencing technology (see accompanying manuscript by Scheijen et al., submitted)

**Supplementary Table 2D: Design of the TRB VJ and DJ panels in consecutive testing rounds**

| **Version** |  | **Time frame** | **Number of FPs** | **Number of RPs** | **Tested reaction conditions** | **Tested clonal controls** | **Tested polyclonal controls** | **Tested neg controls** | **Involved labs** | **Consequence** |
| --- | --- | --- | --- | --- | --- | --- | --- | --- | --- | --- |
| 1 | Initial design | Sept 2012  -Oct 2013 | 49 VB  2 DB | 13 | Primer concentrations: 0.025/0.05/0.1/0.2 µM each  MgCl2: 1.5/3/4/5 mM  Annealing temperature: 54/57/60/63°C  Cycle numbers:  1^st^ PCR: 20/25/30/35 cycles  2^nd^ PCR: 20/25/30/35 | Jurkat cell line | PBMNCs | Aqua  HeLa cell line | Kiel  Berlin | Removal of 10 redundant VB primers  Approval of reaction conditions:  Primer concentrations: 0.025 µM each  MgCl2: 4mM  Annealing temperature: 63°C  Cycle numbers:  1^st^ PCR: 35 cycles  2^nd^ PCR: 20 cycles |
| 2 | Multicentre validation (Pilot 1) | Oct 2013- October 2014 | 38 VB | 13 | Primer concentrations: 0.025 µM each  MgCl2: 4mM  Annealing temperature: 63°C  Cycle numbers:  1^st^ PCR: 35 cycles  2^nd^ PCR: 20 cycles  Gel extraction of second round PCR products | 30 patient samples pool with 42 clonally rearranged TRB  29 cell lines pool with 43 rearranged TRB | Mixed MNCs 20 healthy donors | Aqua  HeLa cell line | Kiel  Torino  Monza  London  Prague | Removal of 2 VB primers  Addition of 1VB primer  Redesign of 7 VB primers  Redesign of 1 JB primer  Adapt primer concentrations |
| 3 | Single-centre validation |  | 38 VB | 13 | Primer concentrations: individualized within multiple testing rounds  MgCl2: 4mM  Annealing temperature: 63°C  Cycle numbers:  1^st^ PCR: 35 cycles | 28 T-cell lines pool (39 rearrangements)  34 patient samples pool (47 rearrangements) |  |  | Kiel | No further modifications |
| 4 | Multicentre validation (ALL marker identification) | Spring 2016-October 2017 | 38 VB  2 DB | 13 | Primer concentrations: individualized  MgCl2: 4mM  Annealing temperature: 63°C  Cycle numbers:  1^st^ PCR: 35 cycles  2^nd^ PCR: 20 cycles | 50 ALL diagnostic samples  Spike-in (9 cell lines pool) | Central polyclonal control (1/3 thymus, 1/3 tonsil, 1/3 PBMNC) | Aqua | Kiel  Prague  London/Bristol  Paris Necker  Monza | Approval of final assay for IG/TR marker identification |

**Supplementary Table 2E: Design of the TRG panel in consecutive testing rounds**

| **Version** |  | **Time frame** | **Number of FPs** | **Number of RPs** | **Tested reaction conditions** | **Tested clonal controls** | **Tested polyclonal controls** | **Tested neg controls** | **Involved labs** | **Consequence** |
| --- | --- | --- | --- | --- | --- | --- | --- | --- | --- | --- |
| 1 | Initial design | Sept 2012-Spring 2014 | 8 | 4 | Primer concentrations: 0.1/0.2 µM each (FPs)  0.2/0.4 µM each (Reverse primers)  MgCl2: 1.5mM  Annealing temperature: 60/63°C  Cycle numbers:  1^st^ PCR: 20/25 cycles  2^nd^ PCR: 30/35 cycles | 6 cell lines pool with 12 rearranged TRG  1 patient samples with 2 clonally rearranged TRG | Mixed PBMNCs 5 healthy donors | Aqua  HeLa cell line | Monza | Redesign of 2 VG primers |
| 2 | Single-centre validation | Spring 2014-Feb 2015 | 8 | 4 | Primer concentrations:  0.1 µM each (FPs)  0.2 µM each  (Reverse primers)  MgCl2: 1.5mM  Annealing temperature: 60/63°C  Cycle numbers:  1^st^ PCR: 25/30cycles  2^st^ PCR: 15/20 cycles  Enzyme: HiFi Fast Start (Roche) / TaqGold (Applied Biosystem) | 27 cell lines pool with 46 rearranged TRG  34 patient samples with 37 clonally rearranged TRG | Mixed PBMNCs 5 healthy donors | Aqua | Monza | Redesign of 1 VG primer |
| 3 | Single-centre validation | Feb 2015-April 2016 | 8 | 4 | Primer concentrations:  0.005/0.2/0.3 µM (FPs)  0.2 µM  (Reverse primers)  MgCl2: 1.5mM/4mM  Annealing temperature: 63°C  Cycle numbers:  1^st^ PCR: 30 cycles  2^st^ PCR: 15 cycles | 27 cell lines pool with 46 rearranged TRG  34 patient samples with 37 clonally rearranged TRG | Mixed PBMNCs 5 healthy donors | Aqua | Monza | Adapt primer concentrations |
| 4 | Multicentre validation (ALL marker identification) | Spring 2016-October 2017 | 8 | 4 | Primer concentrations: individualized  MgCl2: 4mM  Annealing temperature: 63°C  Cycle numbers:  1^st^ PCR: 35 cycles  2^nd^ PCR: 20 cycles | 50 ALL diagnostic samples  cITC (9 cell lines pool) | Central polyclonal control (1/3 thymus, 1/3 tonsil, 1/3 PBMNC) | Aqua | Kiel  Prague  London/Bristol  Paris Necker  Monza | Approval of final assay for IG/TR marker identification |

**Supplementary Table 2F: Design of the TRD panel in consecutive testing rounds**

| **Version** |  | **Time frame** | **Number of FPs** | **Number of RPs** | **Tested reaction conditions** | **Tested clonal controls** | **Tested polyclonal controls** | **Tested neg controls** | **Involved labs** | **Consequence** |
| --- | --- | --- | --- | --- | --- | --- | --- | --- | --- | --- |
| **1** | Initial design | Sept 2012-Oct 2014 | 6 VD  1 DD | 5 JD  1 Ja29  1 DD | Primer concentrations: 0.01/0.1µM each  MgCl2: 1.5/3/4 mM  Annealing temperature: 57/60/63°C  Polymerase : Taq Gold vs Hifi Roche  Cycle numbers:  1Step PCR : 25 or or 30 or 35 cycles | Jurkat, PEER, LOUCY cell line | PBMNCs | Aqua  HeLa cell line | Paris Necker | Removal all VD and addition VD7 and VD8  Approval of reaction conditions:  Primer concentrations: 0.1 µM each  MgCl2: 4mM  Annealing temperature: 63°C  Polymerase : Hifi Roche  Cycle numbers:  1Step PCR : 35 cycles |
| **2** | Single-centre validation | Oct 2014- October 2015 | 8 VD  1 DD | 5 JD  1 Ja29  1 DD | Primer concentrations: 0.1 µM each  MgCl2: 4mM  Annealing temperature: 63°C  Cycle numbers:  1Step PCR: 35 cycles | 18 patient samples pool with 21 clonally rearranged TRD  3 cell lines pool with 6 rearranged TRD | PBMNC of 6 healthy donors | Aqua  HeLa cell line | Paris Necker | Annealing temperature: 62°C |
| **3** | Multicentre validation (ALL marker identification) | Spring 2016-October 2017 | 8 VD  1 DD | 5 JD  1 Ja29  1 DD | Primer concentrations: individualized  MgCl2: 4mM  Annealing temperature: 62°C  Cycle numbers:  1^st^ PCR: 35 cycles  2^nd^ PCR: 20 cycles  AND 1Step PCR comparison | 50 ALL diagnostic samples  cITC (9 cell lines pool) | Central polyclonal control (1/3 thymus, 1/3 tonsil, 1/3 PBMNC) | Aqua | Kiel  Prague  London/Bristol  Paris Necker  Monza | Approval of final assay for IG/TR marker identification |

**Supplementary Table** 3: Primer sequences and primer concentrations. Upper part: 1^st^ step PCR primers. Lower part: 2^nd^ step PCR primers

**Supplementary Table 3** (continued)

**Supplementary Table 4: Patients characteristics including Sanger sequencing IG/TR profiles**

| **Lab** | **Patient ID** | **Immuno-phenotype** | **% blasts**  **at dx** | **IG/TR rearrangements identified by Sanger sequencing** |
| --- | --- | --- | --- | --- |
| Bristol | 10 | T-ALL | 80.0% | - |
| Bristol | 1 | BCP-ALL | 93.6% | IGHV1-2 - IGHD3-16 - IGHJ2 / IGKV1-5 - KDE / TRBD1 - TRBJ2-7 / TRBD1 - TRBJ2-5 / TRBV6-2 - TRBD2 / TRDV2 - TRDD3 / TRDV2 - TRDD3 - TRAJ29 / TRGV9 - TRGJ1 |
| Bristol | 4 | BCP-ALL | 86.0% | IGHD6-13 - IGHJ4 / IGHV3-52 - IGHD3-3 - IGHJ5 / TRDV2 - TRDD3 |
| Bristol | 2 | BCP-ALL | 95.0% | IGHV3-7 - IGHD2-8 - IGHJ6 / TRBV6-1 - TRBD2 - TRBJ2-3 / TRDD2 - TRDD3 / TRDV2 - TRDD3 / TRGV2 - TRGJP2 / TRGV3 - TRGJ1 |
| Bristol | 8 | BCP-ALL | 83.0% | IGHV6-01 - IGHD3-10 - IGHJ6 / IGHV3-43 - IGHD3-10 - IGHJ6 / IGHV2-5 - IGHD2-8 - IGHJ6 / TRBV21-1 - TRBD1 - TRBJ2-5 / TRDV2 - TRDD3 / TRGV4 - J2 / TRGV3 - TRGJP1 |
| Bristol | 7 | T-ALL | 63.0% | TRBD2 - TRBJ2-5 / TRBV21-1 - TRBD2 - TRBJ1-6 / TRBV4-1 - TRBJ2-3 / TRGV2 - TRGJ2 / TRGV8 - TRGJ2 |
| Bristol | 3 | T-ALL | 95.0% | TRBV28 - TRBD1 - TRBJ1-1 / TRBV5-8 - TRBD1 - TRBJ2-5 / TRDD2 - TRDD3 |
| Bristol | 5 | T-ALL | 81.8% | TRBD2 - TRBJ2-1 / TRBD1 - TRBJ1-3 / TRBD2 - TRBJ2-1 / TRBV6-2 - TRBD1 - TRBJ1-3 / TRGV8 - TRGJ1 / TRGV4 - TRGJ1 |
| Bristol | 6 | BCP-ALL | 90.0% | IGHD2-2 - IGHJ6 / IGHD3-3 - IGHJ6 / IGHV4-34 - IGHD2-15 - IGHJ6 / TRBD1 - TRBJ2-7 / TRDD2 - TRDD3 |
| Bristol | 9 | BCP-ALL | 90.0% | IntRSS - KDE / TRDV2 - TRDD3 |
| Kiel | 10 | MPAL | 100.0% | IGHV1-3 - IGHJ4 / IGHV4-31 - J6 / IGKV1-39 - IGKJ5 / TRDV2 - TRDJ1 |
| Kiel | 1 | BCP-ALL | 48.0% | IGHD2-2 - IGHJ6 / TRBD2 - TRBJ2-4 / TRBV13 - TRBJ2-7 / TRDV2 - TRDD3 / TRGV9 - TRGJ1 |
| Kiel | 5 | c-ALL | 67.0% | IGHV3-71 - IGHJ5 / IGKV1-12 - KDE / TRBV10-3 - TRBJ2-3 / TRBV6-5 - TRBJ1-5 / TRDV2 - TRJA29 / TRGV2 - TRGJP2 / TRGV3 - TRGJ1 |
| Kiel | 9 | B-ALL | 90.0% | IGHD2-2 - IGHJ6 / IGHD6-19 - IGHJ6 / IGHV3-30-5 - IGHJ6 / IGHV6-1 - IGHJ6 |
| Kiel | 7 | B-ALL | 90.0% | IGHV3-7 - IGHJ6 / IGKV3-15 - IGKJ1 / TRBV21-1 - TRBJ2-7 |
| Kiel | 4 | c-ALL | 100.0% | IGHV1-2 - IGHJ5 / IGKV2-40 - IGKJ4 / IGKV3-20 - IGKJ4 / IntRSS - KDE (2×) |
| Kiel | 2 | B-ALL | 60.0% | IGHV3-43D - IGHJ6 / IGKV1-8 - KDE / TRBD1 - TRBJ2-5 / TRDD2 - TRDD3 / TRDV2 - TRDD3 - TRDJ2 |
| Kiel | 6 | T-ALL | 80.0% | TRDD2 - TRDJ1 / TRDV2 - TRDD3 |
| Kiel | 8 | T-ALL | 83.0% | IGHD6-19 - IGHJ4 / TRBV7-21 - TRBJ2-1 / TRDV5-TRDJ1 / TRGV4 - J1 / TRGV8 - TRGJ1 |
| Kiel | 3 | c-ALL | 88.0% | IGHV1-2 - IGHJ6 / IGKV2-29 - IGKJ4 / IGKV2-30 - KDE / TRDV2 - TRAJ29 / TRDV2 - D3 / TRGV11 - TRGJ1 / TRGV3 - TRGJ1 |
| Monza | 1 | BCP-ALL | 68.0% | IGHD2-2 - IGHD6-13 - IGHJ6 / IGHV3-13 - IGHJ6 / IGHV4-55 - IGHJ6 / IGKV3-15 - KDE / IGKV1-37 - IGKJ4 / IntRSS - KDE / TRBD2 - TRBJ2-1 / TRBV27 - TRBJ2-5 / TRBV7-2 - TRBJ1-1 / TRDD2 - TRDD3 / TRDV2 - TRDD3 / TRGV2 - TRGJP1 / TRGV11 - TRGJP2 |
| Monza | 10 | BCP-ALL | 90.0% | IGHV3-30 - IGHJ2 / TRBD1 - TRBJ2-7 / TRBV21-1 - TRBJ2-7 / TRDV2 - TRDD3 / TRDV2 - TRDJ1 / TRDD2 - TRDD3 / TRGV8 - TRGJ2 / TRGV9 - TRGJ1 |
| Monza | 2 | BCP-ALL | 90.0% | IGHD4-23 - IGHJ5 / IGHV3-3 - IGHJ4 / TRDD2 - TRDD3 / TRDV2 - TRDD3 |
| Monza | 3 | BCP-ALL | 80.0% | IGHV2-5 - IGHJ6 / IGHV4-55 - IGHJ4 / IGHV3-3 - IGHJ4 / IGKV3-15 - IGKJ4 / TRAV2 - TRAJ29 / TRDV2 - TRDD3 (2×) / TRGV9 - TRGJ2 / TRGV5 - TRGJ2 |

# Supplementary Table 4 (continued)

| **Lab** | **Patient ID** | **Immuno-phenotype** | **% blasts**  **at dx** | **IG/TR rearrangements identified by Sanger sequencing** |
| --- | --- | --- | --- | --- |
| Monza | 4 | BCP-ALL | 94.0% | IGHV3-30 - IGHJ4 / IGKV3-20 - KDE / TRBD2 - TRBJ2-3 / TRDV2 - TRDD3 (2×) / TRGV9 - TRGJ2 |
| Monza | 5 | BCP-ALL | 85.0% | IGHD6-25 - IGHJ4 / IGHV3-30 - IGHJ5 / IGHV2-70 - IGHJ6 / IGKV2-30 - KDE / TRBD1 - TRBJ2-5 / TRDV2 - TRDD3 (2×) / TRGV7 - TRGJ1 |
| Monza | 6 | BCP-ALL | 75.0% | IGHD3-22 - IGHJ5 / IGHV1-3 - IGHJ4 / IGHV3-30 - IGHJ5 / IGKV2-28 - IGKJ5 / TRBD2 - TRBJ2-7 / TRDV2 - TRDD3 / TRDD2 - TRDD3 / TRDV2 - TRAJ29 / TRGV2 - TRGJ1 |
| Monza | 7 | BCP-ALL | 90.0% | IGHD3-22 - IGHJ1 / IGHD7-27 - IGHJ1 / TRRDV1 - TRDJ1 / TRDV3 - TRDJ1 / TRGV9 - TRGJ2 / TRGV3 - TRGJ2 |
| Monza | 8 | T-ALL | 76.0% | TRBD1 - TRBJ1-5 / TRDV1 - TRDJ1 (2×) / TRGV4 - TRGJ2 / TRGV11 - TRGJ2 |
| Monza | 9 | T-ALL | 60.0% | IGHD5-12 - IGHJ4 / IGHD7-27 - IGHJ4 / TRDD2 - TRDJ1 (2×) / TRGV5P - TRGJ1 |
| Paris | 1 | BCP-ALL | 80.0% | IGHD2-15 - IGHJ5 / IGHV1-6 - IGHJ6 / TRBD2 - TRBJ2-7 / TRDV2 - TRAJ29 / TRDV2 - TRDD3 / TRDD2 - TRDD3 |
| Paris | 2 | BCP-ALL | 60.0% | IGHV4-34 - IGHJ5 / TRBD2 - TRBJ2-7 / TRDV2 - TRDD3 / TRDV2 - TRAJ29 |
| Paris | 3 | T-ALL | 90.0% | TRBD2 - TRBJ2-1 / TRBV2-1 - TRBJ2-6 / TRGV5 - TRGJ1-1 / TRGV2 - TRGJP1 |
| Paris | 4 | T-ALL | 40.0% | TRBV4-1 - TRBJ1-1 / TRDV3 - TRDJ1 / TRGV9 - TRGJ1-2 / TRGV2 - TRGJP2 / |
| Paris | 5 | T-ALL | 15.0% | IGHD3-9 - IGHJ4 / IGHD5-5 - IGHJ4 / TRDD2 - TRDJ1 / TRGV3 - TRGJ1 |
| Paris | 6 | T-ALL | 85.0% | IGHD1 - IGHJ4 / IGHD5 - IGHJ3 / TRBD1 - TRBJ1 / TRDV1 - TRDJ1 / TRGV4 - TRGJ1 / TRGV2 - TRGJ1 |
| Paris | 7 | BCP-ALL | 50.0% | IGHV3-64 - IGHJ4 / TRGV2 - TRGJP1 / TRGV8 - TRGJ1-1 |
| Paris | 8 | T-ALL | 95.0% | TRBD1 - TRBJ1-2 / TRBV5-4 - TRBJ2-7 / TRDV1 - TRDJ1 / TRGV10 - TRGJ1 / TRGV9 - TRGJ1 |
| Paris | 9 | T-ALL | 95.0% | IGHD6-19 - IGHJ4 / TRBD2 - TRBJ2-3 / TRBV23-1 - TRBJ2-2 / TRDV1 - TRDJ1 (2×) / TRGV8 - TRGJ1 / TRGV3 - TRGJ1 |
| Paris | 10 | T-ALL | 80.0% | TRBD1 - TRBJ2-3 / TRBV20 - TRBJ2-1 / TRGV9 - TRGJ1 |
| Prague | 1 | BCP-ALL | 80.0% | TRBV10-3 - TRBJ2-7 / TRDV2 - TRAJ29 |
| Prague | 2 | T-ALL | 71.0% | TRBD2 - TRBJ2-3 / TRGV4 - TRGJ2 / TRGV5 - TRGJ2 |
| Prague | 3 | T-ALL | 89.0% | TRBD1 - TRBJ1-3 / TRDV2 - TRDD3 / TRDD2 - TRDJ1 / TRGV4 - TRGJP2 / TRGV4 - TRGJ2 / TRGV10 - TRGJ1 |
| Prague | 4 | BCP-ALL | 79.0% | IGKV3-20 - KDE / intRSS - KDE / TRGV2 - TRGJ1 / TRGV11 - TRGJ1 |
| Prague | 5 | T-ALL | 95.0% | TRDV1 - TRDJ1 / TRGV9 - TRGJ2 / TRGV10 - TRGJ2 |
| Prague | 6 | T-ALL | 88.0% | TRBD1 - TRBJ1-5 / TRGV2 - TRGJ1 / TRGV11 - TRGJ1 |
| Prague | 8 | BCP-ALL | 60.0% | IGHV3-53 - IGHD3-9 - IGHJ4 / TRGV3 - TRGJ2 / TRGV5 - TRGJ1 / TRGV8 - TRGJ2 / TRGV4 - TRGJ2 |
| Prague | 7 | T-ALL | 95.0% | TRDV1 - TRDJ1 / TRDV3 - TRDJ1 / TRGV4 - TRGJ2 / TRGV10 - TRGJ2 |
| Prague | 9 | BCP-ALL | 88.0% | IGHV3-66 - IGHD1-26 - IGHJ4 / IGHV4-38 - IGHD3-9 - IGHJ6 / IGKV1D-12 - KDE / IGKV2-30 - KDE / TRDV2 - TRDD3 |
| Prague | 10 | BCP-ALL | 78.0% | IGHV6-1 - IGHD6-6 - IGHJ3 / IGHV7-4-1 - IGHJ6 / TRVD2 - TRDD3 |

# Supplementary Table 5: % of reads with junction using two purification methods

| **Sample** | **PCR tube** | **Purification method** | **Total reads** | **Reads with junction** | **Reads with junction %** |
| --- | --- | --- | --- | --- | --- |
| 18-00789 | IGH-DJ | AMPure | 69542.00 | 66678 | 95.88% |
| 18-00789 | IGH-VJ | AMPure | 55009.00 | 52343 | 95.15% |
| 18-00789 | IGK-VJ-Kde | AMPure | 72516.00 | 63223 | 87.18% |
| 18-00789 | intron-Kde | AMPure | 24642.00 | 21812 | 88.52% |
| 18-00789 | TRB-DJ | AMPure | 67312.00 | 66304 | 98.50% |
| 18-00789 | TRB-VJ | AMPure | 49631.00 | 48506 | 97.73% |
| 18-00789 | TRD | AMPure | 71901.00 | 58360 | 81.17% |
| 18-00789 | TRG | AMPure | 74489.00 | 74234 | 99.66% |
| 18-00789 | IGH-DJ | GelExtraction | 62912.00 | 60441 | 96.07% |
| 18-00789 | IGH-VJ | GelExtraction | 47274.00 | 46367 | 98.08% |
| 18-00789 | IGK-VJ-Kde | GelExtraction | 92098.00 | 88107 | 95.67% |
| 18-00789 | intron-Kde | GelExtraction | 44371.00 | 44125 | 99.45% |
| 18-00789 | TRB-DJ | GelExtraction | 60580.00 | 60176 | 99.33% |
| 18-00789 | TRB-VJ | GelExtraction | 80718.00 | 80216 | 99.38% |
| 18-00789 | TRD | GelExtraction | 118371.00 | 107508 | 90.82% |
| 18-00789 | TRG | GelExtraction | 88446.00 | 88134 | 99.65% |
