# Supplementary material for: Standardized next-generation sequencing of immunoglobulin and T-cell receptor gene recombinations for MRD marker identification in acute lymphoblastic leukaemia; a EuroClonality-NGS validation study
Source: Leukemia. 2019 Jun 26;33(9):2241–53. doi: 10.1038/s41375-019-0496-7 (PMC6756028; doi:10.1038/s41375-019-0496-7)

**Supplementary information to:**

**Standardized next-generation sequencing of immunoglobulin and T-cell receptor gene recombinations for MRD marker identification in acute lymphoblastic leukemia; a EuroClonality-NGS validation study**

Monika Brüggemann^1*^, Michaela Kotrová^1,2*,^ Henrik Knecht^1^, Jack Bartram^3^, Myriam Boudjogrha^4^, Vojtech Bystry^5^, Grazia Fazio^6^, Eva Froňková^2^, Mathieu Giraud^7^, Andrea Grioni^6^, Jeremy Hancock^8^, Dietrich Herrmann^1^, Cristina Jiménez^9^, Adam Krejci^5^, John Moppett^10^, Tomas Reigl^5^, Mikael Salson^7^, Blanca Scheijen^11^, Martin Schwarz^1^, Simona Songia^6^, Michael Svaton^2^, Jacques JM van Dongen^12^, Patrick Villarese^13^, Stephanie Wakeman^8^, Gary Wright^3^, Giovanni Cazzaniga^6^, Frédéric Davi^4^, Ramón García-Sanz^9^, David Gonzalez^14^, Patricia JTA Groenen^11^, Michael Hummel^15^, Elizabeth A. Macintyre^13^, Kostas Stamatopoulos^16^, Christiane Pott^1^, Jan Trka^2^, Nikos Darzentas^1,5^, Anton W. Langerak^17^, on behalf of the EuroClonality-NGS working group

### Standard operating procedures for IG/TR NGS based marker identification

All standard operating procedures (the version used in this manuscript, as well as the most actual version) are available via the EuroClonality website at www.euroclonality.org/protocols.

### Single-centre validation of IonTorrent as alternative sequencing platforms for EuroClonality IG/TR NGS assays for ALL marker identification.

Reproducibility of the assay was tested on Ion Torrent platform, using the 1st round PCR products of all 8 sequencing libraries created for 10 ALL patients and both positive (cPTC) and negative (aqua) control. Products from the 1st round of PCR except the purified TRB products, were diluted 1:50 unless amplicons were very weak. Primers containing unique sequencing indexes for each reaction tube and Ion Torrent adaptors were used for the 2nd round of PCR. Concentration of the final products was measured using Qubit dsDNA HS Assay Kit and products were pooled into 8 genewise tubes in equimolar ratios before being purified by gelextraction. Purified products were merged in equimolar ratios into one final pool with the final concentration of 90pM and the library was amplified using the Ion OneTouch 2 System. Sequencing was performed on Ion Torrent PGM sequencer with the Ion 318 TM Chip Kit v2 and Ion PGM HiQ Sequencing Kit resulting with 4,547,434 identified reads. Analysis of identified sequences was performed using the ARResT/Interrogate software and further checked with Vidjil software to allow for manual clustering of smaller clones that were the result of homopolymer errors during sequencing. These errors comprise only a minority of analysed reads (median of 2,4% from all analysed reads per target) and the majority of reads allows to identify a marker clonal IG/TR sequence without manual clustering.


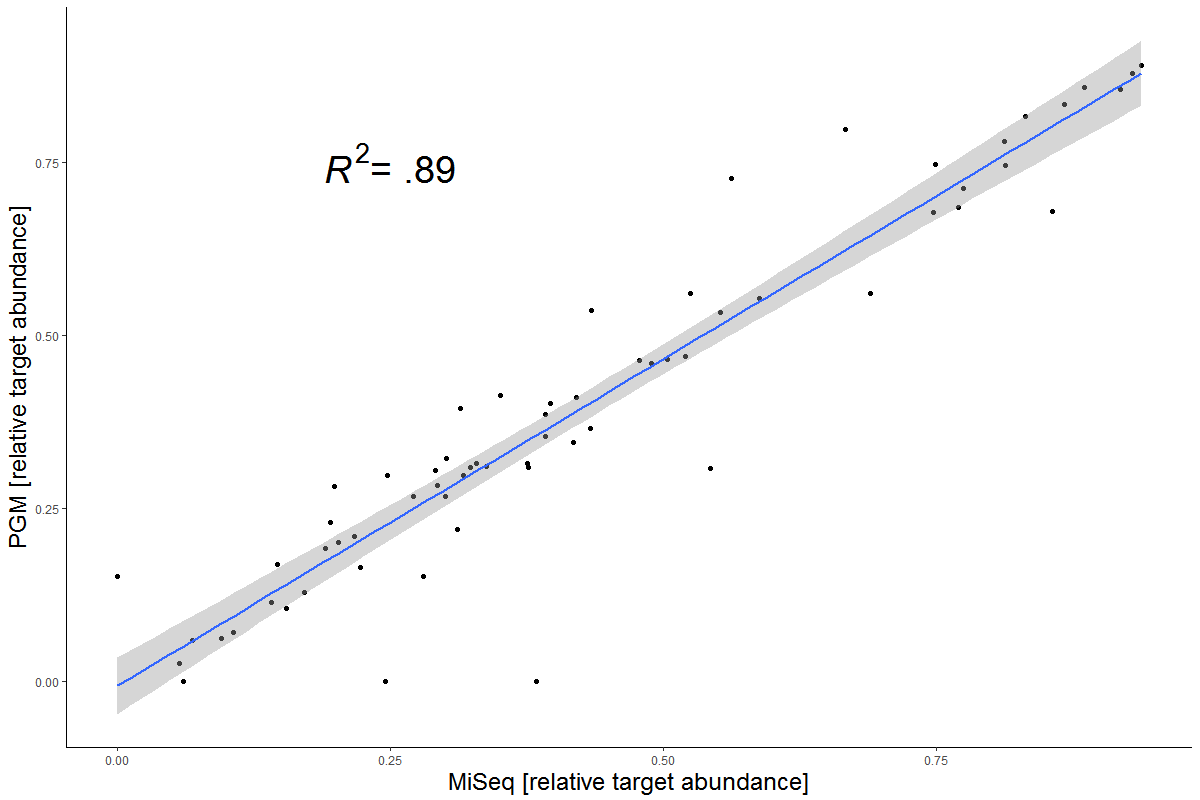
Identified clonal IG/TR sequences were compared to the results of MiSeq data analysis on the basis of their relative abundance based on the number of representing reads. Comparison of the two systems showed that both systems can be used for marker identification with high reproducibility (supp.figure)

***Supplemental figure****. Comparison of relative marker abundances as calculated from both systems. Linear regression model shows reliable correlation of independent results.*

Out of the 65 markers passing the threshold of 5% reads in the tube during data analysis 4 markers would be identified by screening using Illumina MiSeq sequencer that were below the threshold in the data from Ion Torrent. Another marker was only found in the Ion Torrent data above the 5% threshold. The abundance of these markers was commonly around the cut-off threshold and comparable to the polyclonal background and spike-in control.

**Table: Primers for the 2^nd^ PCR containing Ion Torrent adaptors and barcodes.** Those were used with the concentration 0,2µM.

| **Oligo Name** | **Oligo Sequence (5' to 3')** |
| --- | --- |
|  |  |
| trP1MKIE | CCTCTCTATGGGCAGTCGGTGATTAATACGACTCACTATAGGG |
| AMID1 | CCATCTCATCCCTGCGTGTCTCCGACTCAGCTAAGGTAACGATGTAAAACGACGGCCAG |
| AMID2 | CCATCTCATCCCTGCGTGTCTCCGACTCAGTAAGGAGAACGATGTAAAACGACGGCCAG |
| AMID3 | CCATCTCATCCCTGCGTGTCTCCGACTCAGAAGAGGATTCGATGTAAAACGACGGCCAG |
| AMID4 | CCATCTCATCCCTGCGTGTCTCCGACTCAGTACCAAGATCGATGTAAAACGACGGCCAG |
| AMID5 | CCATCTCATCCCTGCGTGTCTCCGACTCAGCAGAAGGAACGATGTAAAACGACGGCCAG |
| AMID6 | CCATCTCATCCCTGCGTGTCTCCGACTCAGCAGAAGGAACGATGTAAAACGACGGCCAG |
| AMID7 | CCATCTCATCCCTGCGTGTCTCCGACTCAGTTCGTGATTCGATGTAAAACGACGGCCAG |
| AMID8 | CCATCTCATCCCTGCGTGTCTCCGACTCAGTTCCGATAACGATGTAAAACGACGGCCAG |
| AMID9 | CCATCTCATCCCTGCGTGTCTCCGACTCAGTGAGCGGAACGATGTAAAACGACGGCCAG |
| AMID10 | CCATCTCATCCCTGCGTGTCTCCGACTCAGCTGACCGAACGATGTAAAACGACGGCCAG |
| AMID11 | CCATCTCATCCCTGCGTGTCTCCGACTCAGTCCTCGAATCGATGTAAAACGACGGCCAG |
| AMID12 | CCATCTCATCCCTGCGTGTCTCCGACTCAGTAGGTGGTTCGATGTAAAACGACGGCCAG |
| AMID13 | CCATCTCATCCCTGCGTGTCTCCGACTCAGTCTAACGGACGATGTAAAACGACGGCCAG |
| AMID14 | CCATCTCATCCCTGCGTGTCTCCGACTCAGTTGGAGTGTCGATGTAAAACGACGGCCAG |
| AMID15 | CCATCTCATCCCTGCGTGTCTCCGACTCAGTCTAGAGGTCGATGTAAAACGACGGCCAG |
| AMID16 | CCATCTCATCCCTGCGTGTCTCCGACTCAGTCTGGATGACGATGTAAAACGACGGCCAG |
| AMID17 | CCATCTCATCCCTGCGTGTCTCCGACTCAGTCTATTCGTCGATGTAAAACGACGGCCAG |
| AMID18 | CCATCTCATCCCTGCGTGTCTCCGACTCAGAGGCAATTGCGATGTAAAACGACGGCCAG |
| AMID19 | CCATCTCATCCCTGCGTGTCTCCGACTCAGTTAGTCGGACGATGTAAAACGACGGCCAG |
| AMID20 | CCATCTCATCCCTGCGTGTCTCCGACTCAGCAGATCCATCGATGTAAAACGACGGCCAG |
| AMID21 | CCATCTCATCCCTGCGTGTCTCCGACTCAGTCGCAATTACGATGTAAAACGACGGCCAG |
| AMID22 | CCATCTCATCCCTGCGTGTCTCCGACTCAGTTCGAGACGCGATGTAAAACGACGGCCAG |
| AMID23 | CCATCTCATCCCTGCGTGTCTCCGACTCAGTGCCACGAACGATGTAAAACGACGGCCAG |
| AMID24 | CCATCTCATCCCTGCGTGTCTCCGACTCAGAACCTCATTCGATGTAAAACGACGGCCAG |
| AMID25 | CCATCTCATCCCTGCGTGTCTCCGACTCAGCCTGAGATACGATGTAAAACGACGGCCAG |
| AMID26 | CCATCTCATCCCTGCGTGTCTCCGACTCAGTTACAACCTCGATGTAAAACGACGGCCAG |
| AMID27 | CCATCTCATCCCTGCGTGTCTCCGACTCAGAACCATCCGCGATGTAAAACGACGGCCAG |
| AMID28 | CCATCTCATCCCTGCGTGTCTCCGACTCAGATCCGGAATCGATGTAAAACGACGGCCAG |
| AMID29 | CCATCTCATCCCTGCGTGTCTCCGACTCAGTCGACCACTCGATGTAAAACGACGGCCAG |
| AMID30 | CCATCTCATCCCTGCGTGTCTCCGACTCAGCGAGGTTATCGATGTAAAACGACGGCCAG |
| AMID31 | CCATCTCATCCCTGCGTGTCTCCGACTCAGTCCAAGCTGCGATGTAAAACGACGGCCAG |
| AMID32 | CCATCTCATCCCTGCGTGTCTCCGACTCAGTCTTACACACGATGTAAAACGACGGCCAG |
| AMID33 | CCATCTCATCCCTGCGTGTCTCCGACTCAGTTCTCATTGAACGATGTAAAACGACGGCCAG |
| AMID34 | CCATCTCATCCCTGCGTGTCTCCGACTCAGTCGCATCGTTCGATGTAAAACGACGGCCAG |
| AMID35 | CCATCTCATCCCTGCGTGTCTCCGACTCAGTAAGCCATTGTCGATGTAAAACGACGGCCAG |
| AMID36 | CCATCTCATCCCTGCGTGTCTCCGACTCAGAAGGAATCGTCGATGTAAAACGACGGCCAG |
| AMID37 | CCATCTCATCCCTGCGTGTCTCCGACTCAGCTTGAGAATGTCGATGTAAAACGACGGCCAG |
| AMID38 | CCATCTCATCCCTGCGTGTCTCCGACTCAGTGGAGGACGGACGATGTAAAACGACGGCCAG |
| AMID39 | CCATCTCATCCCTGCGTGTCTCCGACTCAGTAACAATCGGCGATGTAAAACGACGGCCAG |
| AMID40 | CCATCTCATCCCTGCGTGTCTCCGACTCAGCTGACATAATCGATGTAAAACGACGGCCAG |
| AMID41 | CCATCTCATCCCTGCGTGTCTCCGACTCAGTTCCACTTCGCGATGTAAAACGACGGCCAG |
| AMID42 | CCATCTCATCCCTGCGTGTCTCCGACTCAGAGCACGAATCGATGTAAAACGACGGCCAG |
| AMID43 | CCATCTCATCCCTGCGTGTCTCCGACTCAGCTTGACACCGCGATGTAAAACGACGGCCAG |
| AMID44 | CCATCTCATCCCTGCGTGTCTCCGACTCAGTTGGAGGCCAGCGATGTAAAACGACGGCCAG |
| AMID45 | CCATCTCATCCCTGCGTGTCTCCGACTCAGTGGAGCTTCCTCGATGTAAAACGACGGCCAG |
| AMID46 | CCATCTCATCCCTGCGTGTCTCCGACTCAGTCAGTCCGAACGATGTAAAACGACGGCCAG |
| AMID47 | CCATCTCATCCCTGCGTGTCTCCGACTCAGTAAGGCAACCACGATGTAAAACGACGGCCAG |
| AMID48 | CCATCTCATCCCTGCGTGTCTCCGACTCAGTTCTAAGAGACGATGTAAAACGACGGCCAG |
| AMID49 | CCATCTCATCCCTGCGTGTCTCCGACTCAGTCCTAACATAACGATGTAAAACGACGGCCAG |
| AMID50 | CCATCTCATCCCTGCGTGTCTCCGACTCAGCGGACAATGGCGATGTAAAACGACGGCCAG |
| AMID51 | CCATCTCATCCCTGCGTGTCTCCGACTCAGTTGAGCCTATTCGATGTAAAACGACGGCCAG |
| AMID52 | CCATCTCATCCCTGCGTGTCTCCGACTCAGCCGCATGGAACGATGTAAAACGACGGCCAG |
| AMID53 | CCATCTCATCCCTGCGTGTCTCCGACTCAGCTGGCAATCCTCGATGTAAAACGACGGCCAG |
| AMID54 | CCATCTCATCCCTGCGTGTCTCCGACTCAGCCGGAGAATCGCGATGTAAAACGACGGCCAG |
| AMID55 | CCATCTCATCCCTGCGTGTCTCCGACTCAGTCCACCTCCTCGATGTAAAACGACGGCCAG |
| AMID56 | CCATCTCATCCCTGCGTGTCTCCGACTCAGCAGCATTAATTCGATGTAAAACGACGGCCAG |
| AMID57 | CCATCTCATCCCTGCGTGTCTCCGACTCAGTCTGGCAACGGCGATGTAAAACGACGGCCAG |
| AMID58 | CCATCTCATCCCTGCGTGTCTCCGACTCAGTCCTAGAACACGATGTAAAACGACGGCCAG |
| AMID59 | CCATCTCATCCCTGCGTGTCTCCGACTCAGTCCTTGATGTTCGATGTAAAACGACGGCCAG |
| AMID60 | CCATCTCATCCCTGCGTGTCTCCGACTCAGTCTAGCTCTTCGATGTAAAACGACGGCCAG |
| AMID61 | CCATCTCATCCCTGCGTGTCTCCGACTCAGTCACTCGGATCGATGTAAAACGACGGCCAG |
| AMID62 | CCATCTCATCCCTGCGTGTCTCCGACTCAGTTCCTGCTTCACGATGTAAAACGACGGCCAG |
| AMID63 | CCATCTCATCCCTGCGTGTCTCCGACTCAGCCTTAGAGTTCGATGTAAAACGACGGCCAG |
| AMID64 | CCATCTCATCCCTGCGTGTCTCCGACTCAGCTGAGTTCCGACGATGTAAAACGACGGCCAG |
| AMID65 | CCATCTCATCCCTGCGTGTCTCCGACTCAGTCCTGGCACATCGATGTAAAACGACGGCCAG |
| AMID66 | CCATCTCATCCCTGCGTGTCTCCGACTCAGCCGCAATCATCGATGTAAAACGACGGCCAG |
| AMID67 | CCATCTCATCCCTGCGTGTCTCCGACTCAGTTCCTACCAGTCGATGTAAAACGACGGCCAG |
| AMID68 | CCATCTCATCCCTGCGTGTCTCCGACTCAGTCAAGAAGTTCGATGTAAAACGACGGCCAG |
| AMID69 | CCATCTCATCCCTGCGTGTCTCCGACTCAGTTCAATTGGCGATGTAAAACGACGGCCAG |
| AMID70 | CCATCTCATCCCTGCGTGTCTCCGACTCAGCCTACTGGTCGATGTAAAACGACGGCCAG |
| AMID71 | CCATCTCATCCCTGCGTGTCTCCGACTCAGTGAGGCTCCGACGATGTAAAACGACGGCCAG |
| AMID72 | CCATCTCATCCCTGCGTGTCTCCGACTCAGCGAAGGCCACACGATGTAAAACGACGGCCAG |
| AMID73 | CCATCTCATCCCTGCGTGTCTCCGACTCAGTCTGCCTGTCGATGTAAAACGACGGCCAG |
| AMID74 | CCATCTCATCCCTGCGTGTCTCCGACTCAGCGATCGGTTCGATGTAAAACGACGGCCAG |
| AMID75 | CCATCTCATCCCTGCGTGTCTCCGACTCAGTCAGGAATACGATGTAAAACGACGGCCAG |
| AMID76 | CCATCTCATCCCTGCGTGTCTCCGACTCAGCGGAAGAACCTCGATGTAAAACGACGGCCAG |
| AMID77 | CCATCTCATCCCTGCGTGTCTCCGACTCAGCGAAGCGATTCGATGTAAAACGACGGCCAG |
| AMID78 | CCATCTCATCCCTGCGTGTCTCCGACTCAGCAGCCAATTCTCGATGTAAAACGACGGCCAG |
| AMID79 | CCATCTCATCCCTGCGTGTCTCCGACTCAGCCTGGTTGTCGATGTAAAACGACGGCCAG |
| AMID80 | CCATCTCATCCCTGCGTGTCTCCGACTCAGTCGAAGGCAGGCGATGTAAAACGACGGCCAG |
| AMID81 | CCATCTCATCCCTGCGTGTCTCCGACTCAGCCTGCCATTCGCGATGTAAAACGACGGCCAG |
| AMID82 | CCATCTCATCCCTGCGTGTCTCCGACTCAGTTGGCATCTCGATGTAAAACGACGGCCAG |
| AMID83 | CCATCTCATCCCTGCGTGTCTCCGACTCAGCTAGGACATTCGATGTAAAACGACGGCCAG |
| AMID84 | CCATCTCATCCCTGCGTGTCTCCGACTCAGCTTCCATAACGATGTAAAACGACGGCCAG |
| AMID85 | CCATCTCATCCCTGCGTGTCTCCGACTCAGCCAGCCTCAACGATGTAAAACGACGGCCAG |
| AMID86 | CCATCTCATCCCTGCGTGTCTCCGACTCAGCTTGGTTATTCGATGTAAAACGACGGCCAG |
| AMID87 | CCATCTCATCCCTGCGTGTCTCCGACTCAGTTGGCTGGACGATGTAAAACGACGGCCAG |
| AMID88 | CCATCTCATCCCTGCGTGTCTCCGACTCAGCCGAACACTTCGATGTAAAACGACGGCCAG |
| AMID89 | CCATCTCATCCCTGCGTGTCTCCGACTCAGTCCTGAATCTCGATGTAAAACGACGGCCAG |
| AMID90 | CCATCTCATCCCTGCGTGTCTCCGACTCAGCTAACCACGGCGATGTAAAACGACGGCCAG |
| AMID91 | CCATCTCATCCCTGCGTGTCTCCGACTCAGCGGAAGGATGCGATGTAAAACGACGGCCAG |
| AMID92 | CCATCTCATCCCTGCGTGTCTCCGACTCAGCTAGGAACCGCGATGTAAAACGACGGCCAG |
| AMID93 | CCATCTCATCCCTGCGTGTCTCCGACTCAGCTTGTCCAATCGATGTAAAACGACGGCCAG |
| AMID94 | CCATCTCATCCCTGCGTGTCTCCGACTCAGTCCGACAAGCGATGTAAAACGACGGCCAG |
| AMID95 | CCATCTCATCCCTGCGTGTCTCCGACTCAGCGGACAGATCGATGTAAAACGACGGCCAG |
| AMID96 | CCATCTCATCCCTGCGTGTCTCCGACTCAGTTAAGCGGTCGATGTAAAACGACGGCCAG |

### Optimization of PCR conditions and library purification methods - Removal of polyclonal DNA from reaction mix

DNA of 4 samples with B- and 4 samples with T-cell aplasia after anti-B-/anti-T-cell targeted treatment was used for library preparation. In all these cases aplasia was confirmed by flow cytometry. The two samples with B-cell aplasia were chronic lymphocytic leukemia samples after Rituximab (anti-CD20) treatment and the two samples with T-cell aplasia were T-cell prolymphocytic leukemia samples after Alemtuzumab (anti-CD52) treatment. Spike-in DNA was added to each sample, but no polyclonal DNA was used.

The following figure shows the sequencing libraries after the 2^nd^ PCR and the gel extraction. Negative control (aqua) and polyclonal DNA (BC) were used as negative and positive control, respectively. No excessive primer-dimer formation was observed in any of the PCR tubes.


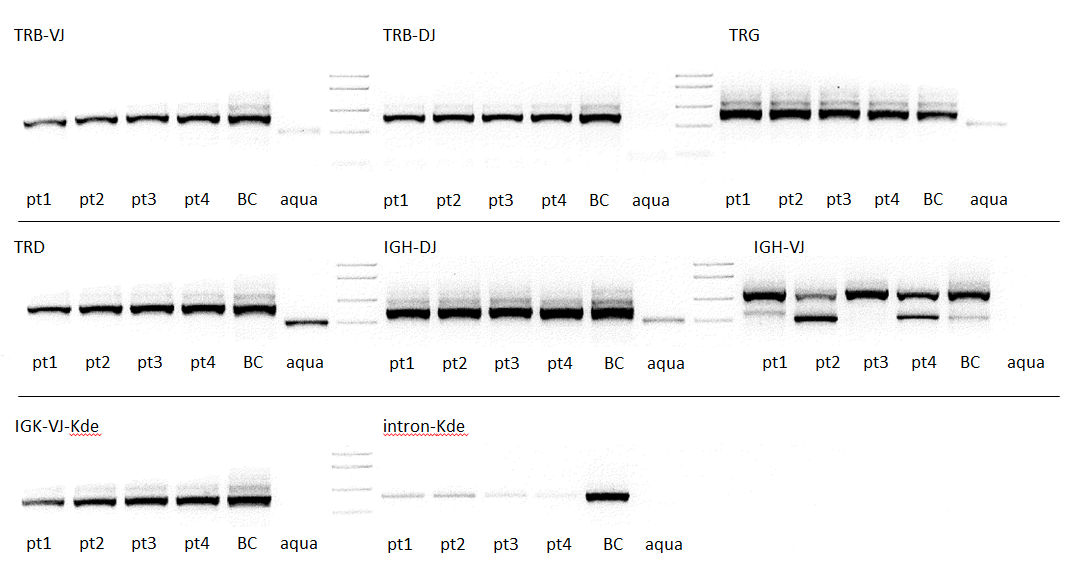

Supplement: Supplementary file 2 — Supplementary Information [file 41375_2019_496_MOESM2_ESM.docx]
